# Prevalence of human papillomavirus infection and associated factors among women attending cervical cancer screening in setting of Addis Ababa, Ethiopia

**DOI:** 10.1038/s41598-024-54754-x

**Published:** 2024-02-19

**Authors:** Elsa Tesfaye, Birhanu Kumbi, Belayihun Mandefro, Yadesa Hemba, Krushna Kant Prajapati, Suresh Chandra Singh, Vijay Upadhye, Sunil Tulshiram Hajare

**Affiliations:** 1 Department of Biology, Faculty of Biomedical Science, College of Natural and Computational Sciences, Dilla University, SNNPR, 419 Dilla, Ethiopia; 2 Shri Krishna Pathology and ELISA Laboratory, Sumerpur, India; 3 TB and COVID Antigen Development Centre, Vadodara, Gujarat India; 4 Research & Development cell, Institute of Applied Sciences,Parul University, Vadodara, Gujarat India

**Keywords:** Cervical cancer, CIN, HPV, PCR, Risk factors, Women, Cancer, Health care, Oncology, Risk factors

## Abstract

Human papillomaviruses (HPVs) are circular, nonenveloped small double-stranded DNA viruses that infect stratified epithelium and can cause a number of life-threatening diseases. HPV is the central risk factor for developing cervical cancer and is estimated that approximately 98% of this disease is associated with oncogenic types of HPV. HPV infection leads to an estimated 266,000 cervical cancer deaths annually. Therefore, the objective of this study was to determine the prevalence of HPV infection and risk factors associated with cervical lesion among women attending the cervical cancer screening clinic at the Ethiopian Family Guidance Association, Addis Ababa. A cross-sectional study was conducted to determine the prevalence of HPV infection. Data were collected using a questionnaire and samples leftover from cervical screening were taken. The leftover swab was air dried and DNA was extracted and amplified by using a PCR. A total of 247 women were included in the study. The prevalence of HPV was 9.72% among the population studied. Of all participants, 27.13% were positive for cervical intraepithelial neoplasia-1 (CIN1). CIN1 positivity was found in half of HPV positive women. Among HPV positive women, half of them had started sexual intercourse at ages 12–17 years and 41.66% were women who gave birth at ages 12–17 years. The high prevalence of HPV and the CIN1 positive group were ages 36–57 and women with multiple sexual partners. The other groups with the highest CIN1 positive were 22.39% grade (9–12) and 20.9% primary (1–8) and uneducated women. Among HPV positive women, 83.33% had an abortion history and 80% miscarried in the first trimester. Among the CIN1 positives, 53.73% had more than two sexual partners. Among HPV positive women, half of them were users of contraception methods. In conclusion, the highest prevalence of HPV is among women who began sexual intercourse earlier and who gave birth at 12–17 years of age, have an abortion history, with MSP and oral contraceptive methods users. In addition to HPV, early pregnancy and sexual intercourse at 12–17 years of age, abortion, MSP, and oral hormonal contraceptives are factors in cervical cancer. Finally, most women do not have enough knowledge and awareness about cervical cancer and the risk factor.

## Introduction

Human papillomaviruses (HPV) are small double-stranded, unenveloped DNA viruses that infect the stratified epithelium^1^. HPV is the central risk factor for developing cervical cancer and is the third most common cancer in women^2–4^.HPV is the most common viral infection of the reproductive tract and it is estimated that approximately 98% of this disease is associated with oncogenic types of HPV^5^. There are more than 200 HPV genotypes present; among the major HRHPV genotypes are HPV16, 18, 31, 33,35, 39, 45, 51, 52, 56, 58, 59, 66 and 68^6,7^. Among those types of oncogenic HPV, 70% of cervical cancer is attributed to HPV16 and HPV18^8^. HPV oncogenicity is primarily dependent on the continuous expression and activity of the viral proteins E6 and E7, which are tumor-associated antigens that act in concert to alter interrelated cellular processes and promote tumor development through the interaction of cellular proteins. Because most HPV infections do not have an obvious clinical symptom, estimates of prevalence and incidence are important to understand the full burden of infection^9^. Although most HPV infections clear up on their own and most pre-cancerous lesions resolve spontaneously, there is a risk for all women that HPV infection can become chronic and pre-cancerous lesions progress to invasive cervical cancer^10^. In Africa, the Americas and Europe, the prevalence of HPV prevalence was observed in the second place among the women aged 45 years or older. Globally, it is estimated that around 291 million women carry HPV DNA, of whom 32% are infected with HPV16 or HPV18, or both^11^. Sexually transmitted infection^12^, hormonal contraceptives^5^, smoking^13^, early sexual intercourse^14^, multiple sexual partners^15^, high parity^16^, early pregnancy^17^ are the risk factors associated with HPV supported by highly suggestive evidence.

**Figure 1:**
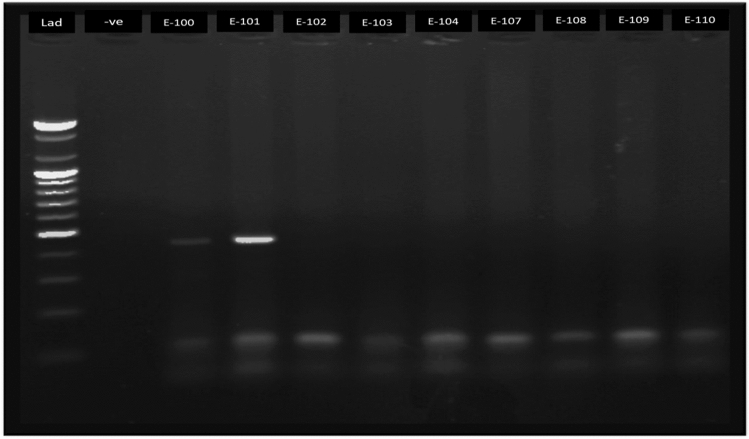
Gel phototograph of MY09/11 PCR product; 109 bp section of human beta globin gene as internal positive control and 450 bp HPV DNA amplicon run with 100 bp DNA ladder and HPV negative control.

The estimated prevalence of HPV in sub-Saharan Africa is 24.4%^18^ and the global prevalence is 11.7%^19^. In Ethiopia, they reported an HPV prevalence of 17.3% and 15.8% for HR HPV^20^. In Addis Ababa, the presence of HPV genotypes in cervical tissues collected from Ethiopian women (*n* = 170) who had cervical cancer was measured using a line probe assay. Of all women studied who had cervical cancer, 93% had HPV DNA in cervical tissues^21^. However, in Addis Ababa there is no data on the prevalence of HPV in the general population (women with and without cervical cancer). In the present study, our objective was to estimate the prevalence of HPV infection and cervical lesion risk factors among women attending cervical cancer screening at the Family Guidance Association of Ethiopia (FGAE) Clinic, Addis Ababa (Fig. 1).

## Materials and methods

### Description of the study area

**Figure 2:**
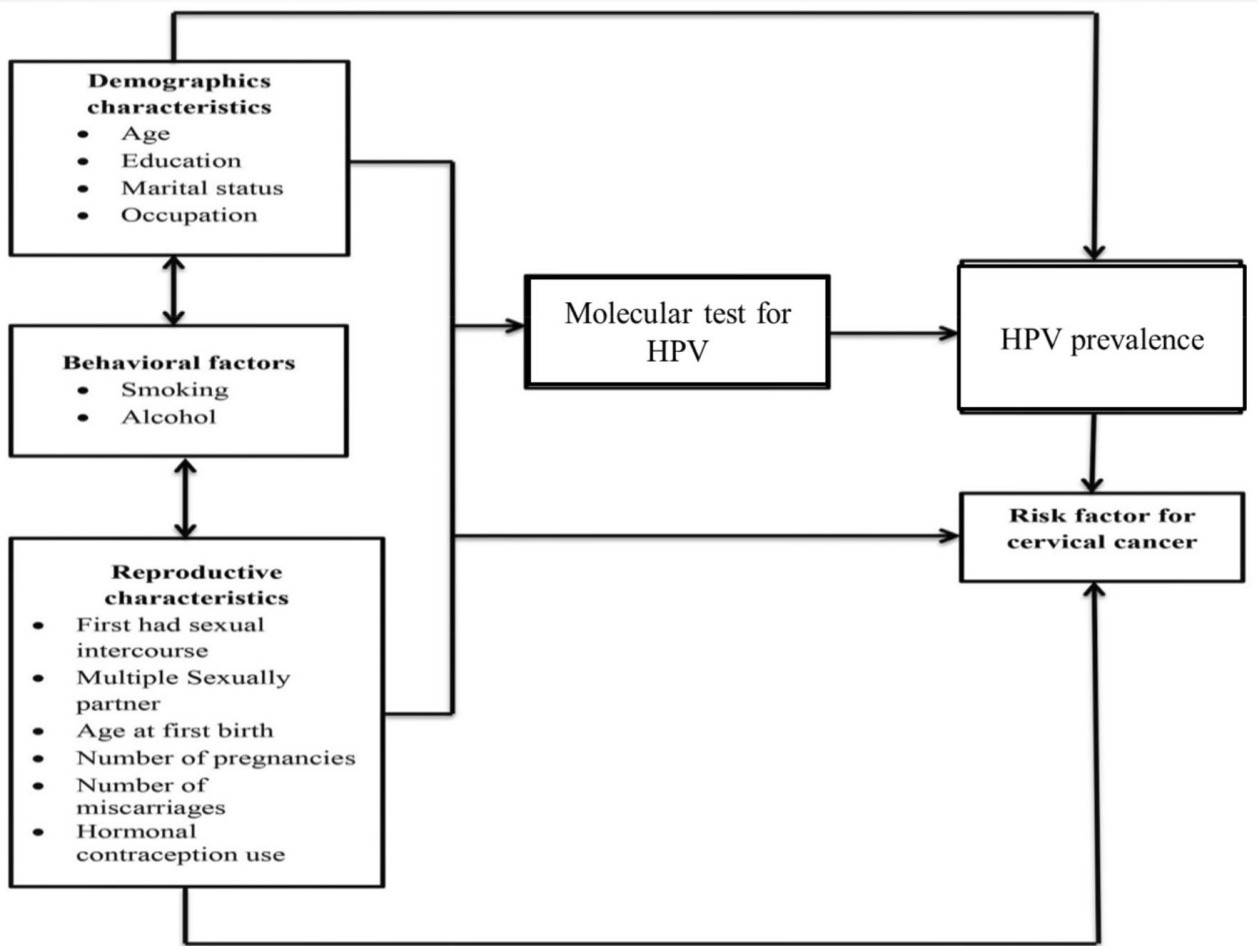
Conceptual frame of the work.

The study was conducted at the FGAE Clinic, Addis Ababa. The FGAE is a network of eight branch offices, seven health clinics, 15 medium sexual and reproductive health clinics, 13 youth centers, ten sex workers' friendly clinics, and one Gynecology and Obstetrics specialty clinic that provides health care services to the community and mainly focused on family planning and reproductive health services. The conceptual framework work of this study is provided in Fig. 2.

### Study design and period

A cross-sectional study was conducted to determine the prevalence of HPV infection among women who attended cervical cancer screening at the FGAE Clinic in Addis Ababa. The study was carried out between September and October 2021. The study involved the administration of a structured questionnaire to women seeking cervical screening in the FGAE. The women were asked for their consent to participate, and leftover samples were taken for HPV testing. Cervical swabs were taken from endocervical regions. The study was approved by the appropriate institution (Addis Ababa City Administration Health Bureau, Ref. HBA/ CSt45/21) and all methods were performed in accordance with the relevant guidelines.

### Subjects

Reproductive age women who came to cervical cancer screening during September and October 2021 who were willing to participate in the study.

**Inclusion criteria**

All women of reproductive age (16–57 years) who attended cervical cancer screening and were willing to participate in the study were included.

**Exclusion criteria**

Study participants absent between September and October and unwilling to participate in the study were excluded from the study. Women who did not undergo a gynecological examination or had been hysterectomized were also excluded.

### Sampling size and sample technique

#### Sample size

The sample size was calculated using a single population proportion formula considering a 5% error margin, a 95% confidence level (CI), 17.3% an age-standardized prevalence of HPV in Ethiopia^20^ and a 10% nonresponse rate. Accordingly, the final sample size is **242**.

The sample size (n) was determined using the following statistical formula.

Sample size for the prevalence of HPV

$${\text{n}} = \frac{{{\text{Z}}^{2} .{\text{P}}\left( {1 - {\text{P}}} \right)}}{{{\text{d}}^{2} }}$$

d = margin of error between the sample and the population. n = sample size. Z = 95% confidence interval. P = prevalence rate of HPV and *C. trachomatis* based on the previous study.

$$\begin{gathered} {\text{n}} = \frac{{1.96 \times 0.173\left( {1 - 0.173} \right)}}{{\left( {.05} \right)^{2} }} \hfill \\ {\text{n}} = \frac{3.84 \times 0.143}{{0.0025}} = 0.54/0.0025 = 220 \hfill \\ \end{gathered}$$

By adding 10% for non- response, the final sample size was 242.

#### Sampling technique and data collection

A consequential sampling technique was used. The main instruments used to collect data for this study were questionnaire and samples leftover from cervical screening were taken for laboratory analysis of infection detection. Sociodemographic characteristics, reproductive characteristics, and behavioral factors were collected using a structured questionnaire. It was used to obtain information on age, occupation, educational background and fertility status, age at marriage or first sexual intercourse, number of sexual partners and use of contraception. Participants who could not read or write were interviewed in Amharic by the researcher. The original questionnaire which was prepared in English was translated into Amharic version to avoid language barriers. Cervical swab was collected from all participants. The clinical examination was performed by trained nurses or midwives and cervical specimens were collected. The swab was taken for HPV detection after being smeared onto glass slides for routine cytology. The endocervical swab was air-dried until further analysis of the HPV DNA using PCR.

#### DNA extraction

DNA extraction was performed with minor modifications to the manufacturer’s protocols (MGI HPV DNA extraction kit-f, China). The dried endocervical swab was immersed in a 1.2 ml lysis solution containing 12 µL of 20 mg / ml proteinase K and incubated at 55 °C for 6 h (and was kept at room temperature overnight if necessary). The swab was removed. 650 µL Cold solution of 8 M ammonium acetate with 1 Mm ethylene diamine tetraacetate (EDTA) was added and mixed gently vortexing. The sample was chilled on ice for about 10 min. For 20 min, the sample was centrifuged at 6000 rpm. 750 µL of the supernatant were transferred to two clean 1.5 ml microtubes containing 750 µL cold isopropanol with wide-bore pipette tips and mixed by inverting gently several times (20 times). After centrifuging the sample at 13,000 rpm for 10 min, the supernatant was poured off and each tube inverted. The pellets were left to drain on clean absorbent paper. 1 ml of 99% ethanol was added and mixed by gently inverting it several times. The samples were centrifuged for 10 min at 13,000 rpm, and the ethanol was poured off and each tube inverted before being drained onto clean absorbent paper. The pellets were resuspended in 100 µL 1XTE buffer (10 mM Tris and 1 mM EDTA, pH 8) and stored at − 4 °C until analyzed. The quantity of DNA was checked using the absorbance of UV light by a NanoDrop spectrophotometer (NanoDrop Thermo Scientific 2000). The extracted DNA was then stored at -4 °C until use.

#### PCR component and profile

DNA extracted from all samples were tested for the presence of HPV DNA using the degenerate primers MY09 (5 ‘CGTCCMARRGGAWACTGATC 3 ‘) and MY11 (5 ‘GCMCAGGGWCATAAYAATGG 3 ‘) as forward and reverse primers. The amplification of the humanbeta globin gene segment with primers Hbtgl1 (5' ACACAACTGTGTTCACTAGC 3') and Hbtgl2 (5 ‘CAACTTCATCCACGTTCACC 3 ‘) as forward and reverse was also multiplexed to serve as internal control of DNA quality and PCR success (Fig. 1). The PCR components and composition were 0.15 µL of MY09 and MY11 primers, 0.07 µL Hbtgl1 and Hbtgl2 primer, 0.5 µL dNTPs, 1 µL of 2 mM MgCl2 solution, 1 µL BD PCR buffer, 0.3 µL Taq polymerase, and 6.06 µL nuclease free water in 10 µL reaction mix. The PCR profile was; initial denaturation at 95 °C for four minutes followed by 35 cycles of denaturation at 95 °C for 30 s, annealing at 50 °C for 45 s, and extension (polymerization) at 72 °C for 45 s and final extension at 72 °C for 7 min. Upon gel-electrophoresis the presence of 450 bp band was interpreted as HPV positive and the 110 bp band as PCR success and the presence of human (internal PCR control). All DNA samples were expected to have the 110 bp band unless the PCR process failed, but only HPV DNA positive samples have the 450 bp band (Supplementary file S1; Fig. 1).

#### Data analysis and interpretation

The generated data was coded and entered using EpiData Manager Version 1.4.4.0. EpiData Manager Date was exported to Microsoft Excel 2010 and imported data exported into Rstudio. Analysis was performed with statistical packages within R version 4.1.2. Data were analyzed using descriptive statistics to describe the characteristics of the study population. Difference in HPV positivity rates between screening results analyzed by the Chi-square test (χ2) and the* F* test. The Pearson correlation test was performed on factors associated with HPV and CIN1. *P* values less than 0.05 were considered statistically significant.

### Ethics approval and consent to participate

The study was carried out following ethical approval obtained from the Addis Ababa City Administration Health Bureau (Ref. HBA/ CSt45/21). The permission of the FGAE Clinic was sought before initiating data collection by communicating with the medical director ‘s office through official letters from Dilla University (DU.PG Biomed 554/21). Written informed consent was obtained from each reproductive age woman to be included in the study and was informed of their rights to interrupt participation at any time. The participants were participating on a voluntary basis. The consent form was read in the local language and a copy was given to the women on request. To ensure confidentiality, participants' data were linked to a code number and the data collection procedure was anonymous; participants' names and any identifiers were not written on the questionnaire and also during the interview, and they were interviewed alone to protect their privacy.

## Results

### Demographic characteristics of the participant

A total of 247 women attended cervical cancer screening at the FGAE clinic during the study period. The majority of the respondents were in age groups of 36–57 years, 212 (85.83%). 62.75% of the participants were married and the majority of 73 (29.55%) of the participants attended grade 9–12, 132 (53.44%) of the participants were unemployed, and 159 (64.37) of the participants earn more than 3000 Birr per month (Table 1, Supplementary File S2).

**Table 1:** Socio-demographics characteristics of women attending cervical cancer screening at FGAE clinic, Addis Ababa (Sep—Oct, 2021).

Characteristics		Frequency *n* = 247 (%)
Age	16–25	2(0.81%)
	26–35	33(13.36%)
	36–57	212(85.83%)
	Divorced	38(15.38%)
Marital status	Married	155(62.75%)
	Single	20(8.1%)
	Widowed	34(13.77%)
	1stDegree and above	27(10.93%)
	Diploma	30(12.15%)
Educational status	Certificate	12(4.86%)
	Grade9-12	73(29.55%)
	Primary(1–8)	61(24.7%)
	Read and write	10(4.05%)
	Illiterate:	34(13.76%)
	Employed full-time	82(33.2%)
Employment status	Employed part-time	28(11.34%)
	Student	5(2.02%)
	Unemployed	132(53.44)%
	More than 3000 Birr	159(64.37%)
Monthly income	1501-3000Birr	41(16.6%)
	501-1500Birr	35(14.17%)
	500and less Birr	12(4.86%)

### Behavioural and reproductive characteristics of the participant

Of the study participants, 115 (46.56%) had started sexual intercourse at 12–17 years of age and among those 75 (65.21%) used alcohol and 78 (67.83%) used contraceptive methods. Among participants, 166 (67.21%) of the participants used contraceptive methods and 54 (32.53%) used pills. Among the participants, 133 (53.85%) had an abortion history; among those, 61 (45.86%) had started sexual intercourse between the ages of 12 and 17. Among those who had an abortion history, most 95 (71.43%) aborted at 1–3 months (first trimester). Of all the participants, only 145 (58.7%) of them had been diagnosed with cervical cancer prior to the survey (Table 2).

**Table 2:** Behavioral and reproductive characteristics of Woman.

Characteristics					Frequency *n* = 247 (%)	
Alcohol consumption	Yes		Per day		6(2.43%)	158–63.97%
			Per week		42(17%)	
			Per month		42 (17%)	
			During festival		68 (27.53)	
	No				89 (36.03)	
Tobacco	Yes		More than 5 year 1–3 year		2 (0.81%)	4 (1.62%)
			3–6 Month		1 (0.40%)	
					1 (0.40%)	
	No				243 (98.97%)	
	Smoke tobacco now		Yes		2 (0.81%)	
			No		2 (0.81%)	
Sex	AAFSI		12–17		115 (46.56%)	
			18–23		99 (40.08%)	
			Above 24		33 (13.36%)	
	Number of Sexual partners		More than 4		25 (10.12%)	
			2–4		105 (42.51%)	
			1		117 (47.37%)	
Contraceptive use	Yes		-Pills		54 (21.86%)	166 (67.21%)
			-Injectable Depo		30 (12.15%)	
			-IUD (loop)		25 (10.12%)	
			-Implant		14 (5.67%)	
			-Pills, Injectable Depo, IUD(loop)		18(7.29%)	
			-Pills, Implant, IUD (loop)		10 (4.05%)	
			-Pills, Implant and Injectable Dep		10 (4.05%)	
			-Condom		2 (0.80%)	
			-Emergency contraceptive		3 (1.21%)	
	No				81 (32.79%)	
Parity			12–17		98 (39.68%)	235 (95.14%)
			18–23		82 (33.2%)	
	Yes	AAFP	Above 24		55 (22.27%)	
			Number of children	1–3	162(65.59%)	
				4–6	61 (24.69%)	
				7–12	12 (4.86%)	
	No				12 (4.86%)	
Abortion	Yes		1–3 month (first trimester)		95 (38.46%)	133 (53.85%)
			First and second trimester		17 (6.88%)	
			First and third trimester		1 (0.40%)	
			4–6 month (Second trimester)		11 (4.45%)	
			7–9 month (Third trimester)		4 (1.62%)	
			Second and Third trimester		1 (0.40%)	
			All trimester		4 (1.62%)	
	No				114 (46.15%)	
Screening history	Yes				145 (58.7%)	
	No				102 (41.3%)	

### Prevalence of HPV

The general prevalence nonspecific HPV test with MY09/11 PCR was 24 (9.72%). The most HPV positive group was age 36 and older than 21 (8.5%) and there was no HPV prevalence between 16 and 25 years. The prevalence of HPV among women with grade 9–12 education status is 3.24% (*n* = 8) and primary (1–8) 2.83% (*n* = 7). Furthermore, women who are married 5.67% (*n* = 14), those who used alcohol during the festival 2.83% (*n* = 7), AAFP 12 -17 4.05% (*n* = 10), multiple sexual partners 2–4 4.86% (*n* = 12), and abort in the first trimester 1–3 6.48% (*n* = 16) had a high prevalence of HPV (Table 3).

**Table 3:** Prevalence of HPV among women.

Characteristics				HPV		p-value
				Negative	Positive	
Alcohol	Yes		Perdays	6	0	0.9465
			Perweak	39	3	
			Permonth	37	5	
			Duringfestival	61	7	
	No			80	9	
Tobacco	Yes		Morethan5year	2	0	1
			1-3 year	1	0	
			3–6 Month	1	0	
	No			219	24	
Sex	AAFSI		12–17	103	12	0.3484
			18–23	92	7	
			Above24	28	5	
	Sexualpartner		Morethan 4	22	3	0.5456
			2–4	93	12	
			1	108	9	
Contraceptive use	Yes		-Pills	49	5	0.5291
			-InjectableDepo	28	2	
			-IUD(loop)	24	1	
			-Implant	14	0	
			-Pills,InjectableDepo,IUD(loop)	16	2	
			-Pills,Implant,IUD(loop)	10	0	
			-Pills,ImplantandInjectableDep	9	1	
			-Condom	2	0	
			-Emergencycontraceptive	2	1	
	No			69	12	
	Yes	AAFP	12–17	88	10	0.9944
Parity			18–23	74	8	
			Above24	50	5	
	No			11	1	
Abortion	Yes		-1-3firsttrimester	79	16	0.0209
			-Firstandsecondtrimester	16	1	
			-Firstandthirdtrimester	1	0	
			-4-6Secondtrimester	9	2	
			-7-9Thirdtrimester	3	1	
			-Secondandthirdtrimester	1	0	
			-Alltrimester	4	0	
	No			110	4	
Screeninghistory	Yes			131	14	1
	No			92	10	

### Factors associated with HPV prevalence

**Figure 3:**
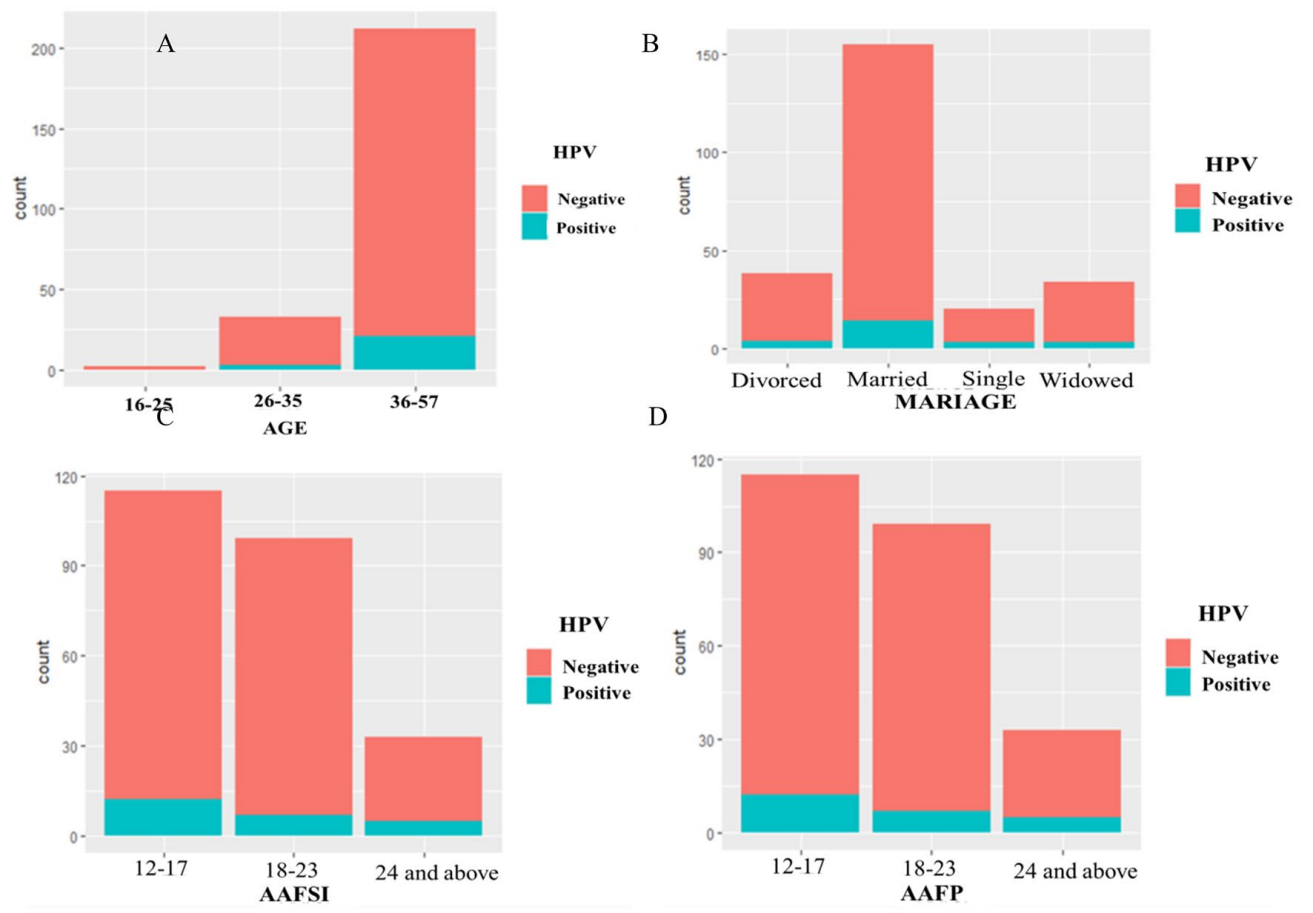
HPV prevalence by (**A**) Age; (**B**) Marriage; (**C**) AAFSI; (**D**) AAFP.

Among HPV positive women, 83.33% (*n* = 20) had a history of abortion, and among those 80% (*n* = 16) aborted during the first trimester (1–3 months). Of all participants, 53.85% (*n* = 133) had an abortion history and most of them 70.68% (*n* = 94) used contraceptive methods. Among women who had an abortion history, 85.71% (*n* = 114) were between the ages of 36 and 57 years. Among HPV positive women, 87.5% (*n* = 21) were between the ages of 36 and 57 years. Among the 235 women who had children, 86.81% (*n* = 204) were between the ages of 36 and 57 and among those 41.18% (*n* = 84) gave birth between the ages of 12 and 17. Among HPV positive women, 41.66% (*n* = 10) were women who had given birth at age 12–17. Among 115 women who began sexual intercourse at 12 to 17 years of age, 85.22% (*n* = 93) gave birth at 12 to 17. And also 32.17% (*n* = 37) and 27.83% (*n* = 32) attended primary education (1–8) and grade 9–12, respectively. Among HPV positive half of them 50% (*n* = 12) started sexual intercourse between 12 and 17 years old and 33.33% (*n* = 8) and 29.17% (*n* = 7) attended grade 9–12 and primary (1–8) school respectively. Women who started sexual intercourse at 12 to 17 years of age (26.32% (*n* = 65) and among HPV positive 62.5% (*n* = 15) had more than 2 sexual partners (Fig. 3).

### Factors associated with cervical screening results

**Figure 4:**
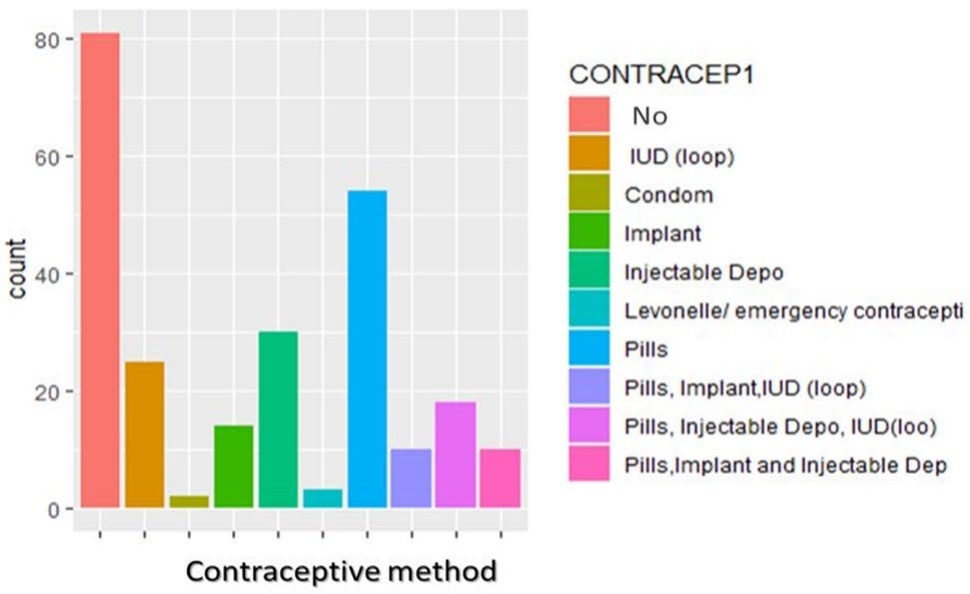
Contraceptive users among women.

**Figure 5:**
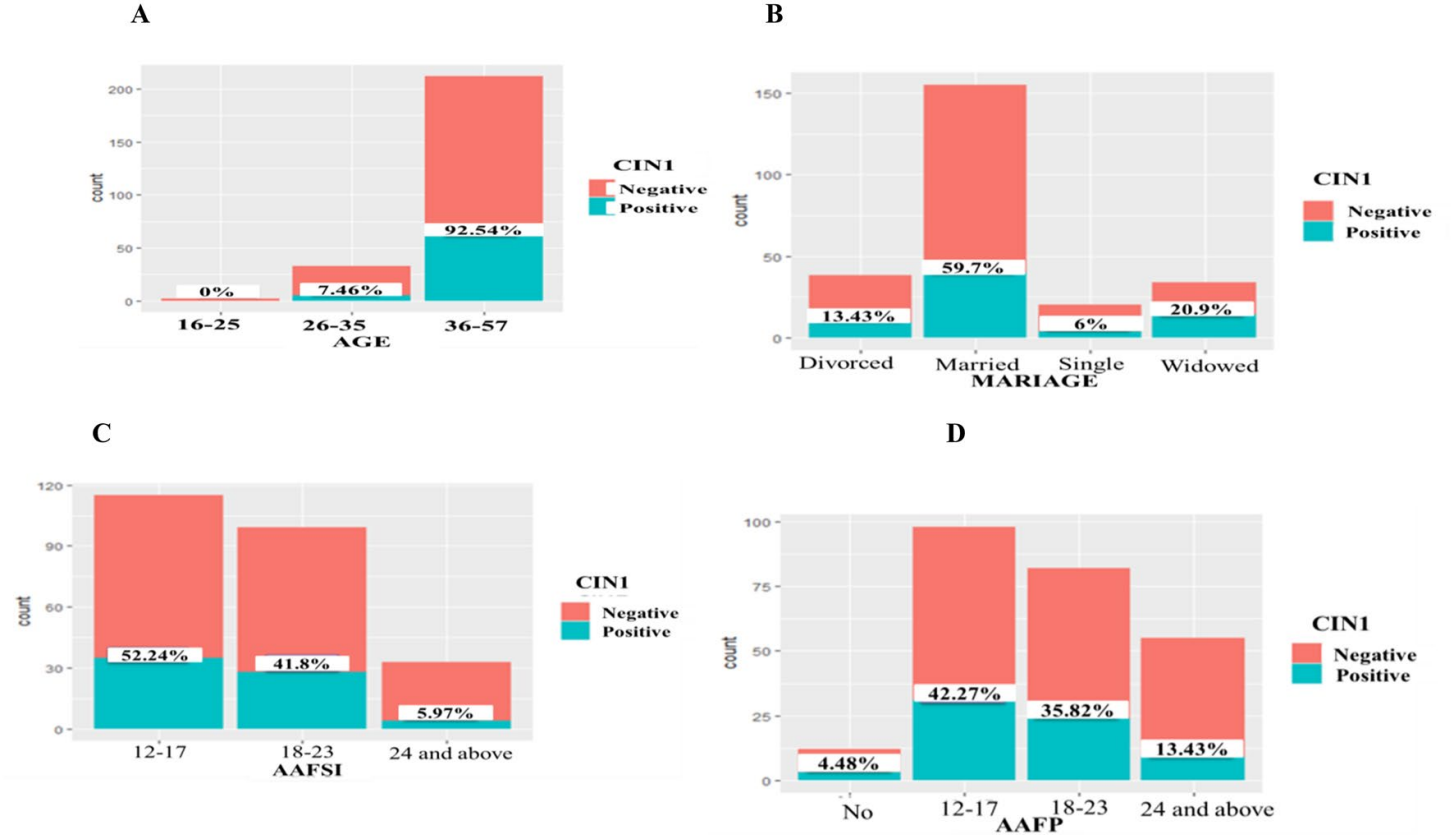
CIN1 positive among (**A**) Age group; (**B**) Marital status; (**C**) AAFSI; (**D**) AAFP.

Of all participants, 67 (27.13%) participants had a positive result in cervical cancer screening (Pap test). Among CIN1 positive women, 12 (17.91%) were HPV positive. Among CIN1 positive women, the most vulnerable group to CIN1 was age 36–57 years 62 (92.54%). Among CIN1 positive women, 14 (20.9%) were married. Among women who are married, 117 (85.40%) used contraceptive methods and 60 (51.28%) used pills (Fig. 4). The other groups with the highest CIN1 positive were 22.39% grade (9–12) and 20.9% primary (1–8) and uneducated women. And also 52.24% of AAFSI at 12–17 years of age and 46.27% of AAFP at 12–17 years of age were CIN1 positive. Among women with AAFP at 12–17 years of age, 51.02% had an abortion history and 70% aborted during the first trimester (1–3 months). Among CIN1 positives, 64.18% used alcoholic beverages and 53.73% had more than two sexual partners. There was no CIN1 positivity among smokers. CIN1 positivity was found in half of HPV positive women (50%) (Fig. 5).

## Correlation of the variables

### Correlation of the sociodemographic factors

**Figure 6:**
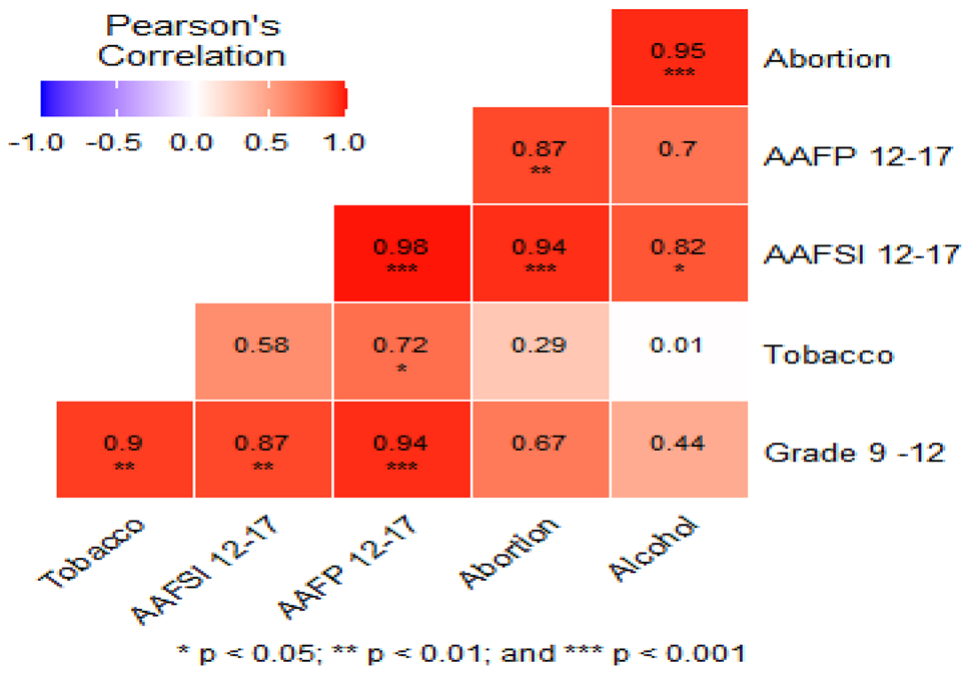
Pearson ‘s correlation of AAFSI 12–17, AAFP 12–17, Abortion, tobacco, alcohol and grade 9–12.

Pearson ‘s correlation of risk factors showed the statistical association between the variables; AAFSI 12–17, AAFP 12–17, Abortion, tobacco smoking, alcohol use and education for grades 9–12, had a significant correlation with each other depending on the number of CIN1 and HPV result. All risk factors (AAFSI 12–17, AAFP 12–17, abortion, tobacco smoking, alcohol use, and grade 9- 12 education) had a positive correlation. Abortion had a positive correlation of 0.94 (p < 0.001) and 0.87 (p < 0.01) with AAFSI 12–17 and AAFP 12–17, respectively. AAFSI 12–17 had a positive correlation of 0.98 (*P* < 0.01) and 0.87 (*P* < 0.01) with AAFP 12–17 and grade 9–12, respectively. Tobacco had a positive correlation 0.9 (*P* < 0.01) and 0.1 (*P* < 0.05) with grade 9–12 and alcohol, respectively (Fig. 6).

### Correlation of the variables, CIN1, and HPV

According to an odd ratio, women with HPV were 3.05 times more likely to develop CIN1 than those women with a negative HPV result. Women with an abortion history were 4.87 times more exposed to HPV than women without an abortion history (Table 4).

**Table 4:** Odd ratio and *P* values of CIN1, HPV, contraception and abortion.

	CIN1	HPV	Contraception
HPV	*P* = 0.01388;		
	OR = 3.05 (1.30, 7.19)		
Contraception	*P* = 0.7623	*P* = 0.06894	
	OR = 0.91 (0.50, 1.65)	OR = 0.45 (0.19, 1.05)	
Abortion	*P* = 0.886	*P* = 0.004594	*P* = 0.2235
	OR = 1.08 (0.61, 1.90)	OR = 4.87 (1.61, 14.70)	OR = 1.41 (0.83, 2.40)

### Knowledge about risk factors for cervical cancer

**Figure 7:**
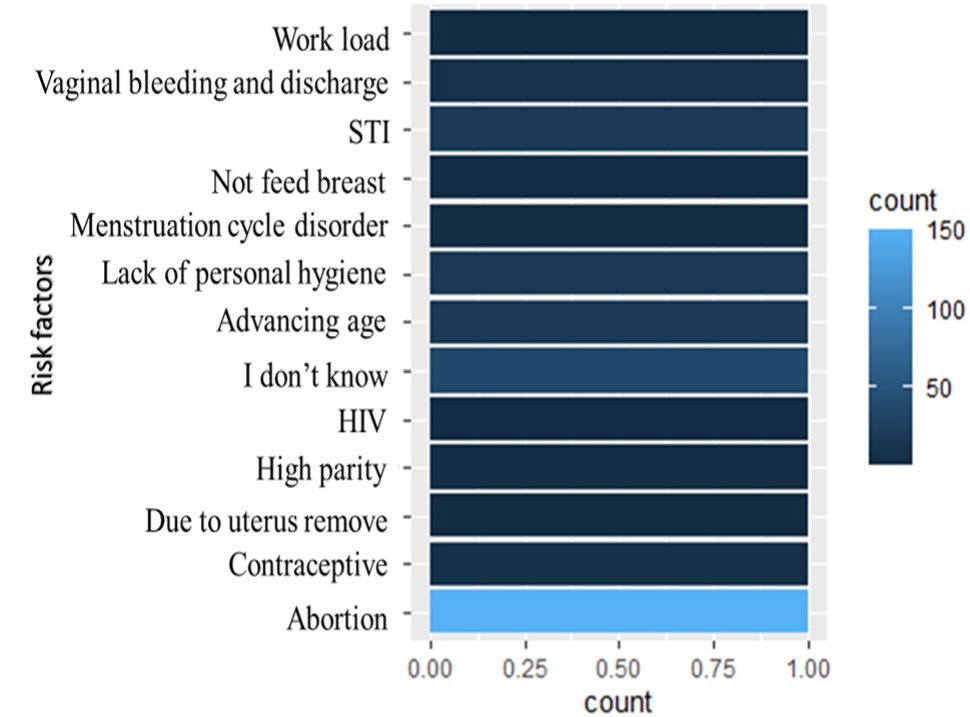
Women ‘s knowledge about risk factors for cervical cancer.

In general, most women believe that 20 (8.1%) advancing age and STI, 150 (60.73%) abortion, 8 (3.24%) vaginal bleeding and discharge, 7 (2.83%) contraception and 19 (7.7%) lack of personal hygiene were risk factors for cervical cancer. Some 7 (2.83%) believe HIV, menstruation cycle disorder, breast not feeding, and work load were risk factors for cervical cancer. The remaining 47 (19.03%) do not know about risk factors for cervical cancer. No one could mention HPV infection as risk factors for cervical cancer (Fig. 7).

## Discussion

This study aims primarily to evaluate the prevalence of HPV, along with potentially associated sociodemographic, behavioral, and reproductive variables, in FGAE clinics in Addis Ababa, Ethiopia. The general prevalence in this cross-sectional population study in women was 9.72%, which is consistent with reports from Asia (9.4%)^18^, but almost twice as low as in other African countries (21.1%)^22^. In a study in northern Africa, a Muslim community, the prevalence of HPV was 6.3%^23^ slightly lower than our study. The overall prevalence of abnormal cytology (CIN1) was 27.13%. Women with HPV were 3.05 (1.30, 7.19) times (*P* = 0.01388) more likely to develop CIN1 than women who had a negative HPV result. Globally, among women with normal cervical cytology, HPV prevalence was 9.9% in 2019^22^. However, in this finding, the prevalence of HPV among normal cytology was 4.86%, which is lower than global reports. In the Amhara region, the prevalence of HPV among women without cervical lesions was 2.4%^24^, which is less than in these studies. Among married women, more than half use oral contraceptives. The prevalence of HPV was higher in oral contraceptive users than in other contraceptive users^25^. In contrast, Castle et al.^16^ reported that hormonal contraceptives had little or no impact on the development of HPV infection or on the development of CIN. Age-specific variation in HPV prevalence has been reported globally. Ages 36 to 57 years were the most CIN1 and HPV positive group in the current study. An older age can influence epigenetic changes that can influence the gene expression of the host's cellular genome. In cervical cancer, epigenetic changes play an important role in its development^26,27^. Older women are more likely to develop persistent HPV infections and cervical lesions, as reported in several previous studies^24,26,27^. In our study, HPV infection was not detected among the ages 16–25 years. This is in contrast to previous studies which reported that there was a high peak of HPV prevalence among younger women, which decreased with age^28,29^. This may be due to fewer participants in the age group of 16–25 years enrolled in our study.

We observed that women who initiated the first sexual relationship at the age of 12–17 years were at increased risk of developing CIN1 and HPV prevalence. Among HPV positive individuals, half of them started sexual intercourse at 12–17 years of age. Similarly, in a study in Bahir Dar Town, women who had initiated sexual intercourse at the age of 18 were 1.7 times more likely to have the lesion compared to those who initiated sexual intercourse at the age of 18 years and later^30^. Starting sexual intercourse at an earlier age increases the risk of HPV and cervical cancer, as reported in several previous studies^14,30,31^. Having started sexual intercourse at an earlier age leads women to give birth before the age of 18 years. Among HPV positive women, 41.66% were women who gave birth at 12–17 years of age. In addition to early age at first sexual intercourse, early childbirth has also been linked as a risk factor for cervical carcinogenesis and attributed to cervical trauma experienced during early age at first pregnancy. Cervical trauma also increases the persistence of HPV infection^32^. In our study, more than half of women with multiple sexual partners (MSP) were CIN1 positive and had a high prevalence of HPV. This dominant risk factor for MSP has been documented in several previous studies^33–35^.

Our findings on the history of abortion among HPV positive women are consistent with those found in Swaziland^36^ and Colombia^37^. In our study, abortion had a positive correlation of 0.94 (*P* < 0.001) and 0.87 (*P* < 0.01) with AAFSI 12–17 and AAFP 12–17, respectively. Women with an abortion history were 4.87 (1.61, 14.70) times more likely (P = 0.004594) to have HPV infection than women who did not have an abortion history. We found that women who smoked tobacco had no positive result with HPV or CIN1. We could not accurately estimate the impact of smoking on HPV prevalence because very few participants had a smoking habit and were not ready to tell the truth due to cultural influence. Although a previous study reported that smoking habit 14 (7.82–25.32) times increased the risk of cervical cancer development than nonsmokers^13,38^. Most women do not have sufficient knowledge and awareness about cervical cancer and risk factors for precancerous lesion. Lack of knowledge is a risk factor for the prevalence of HPV and precancerous lesion. Most of the participants believed that the causes of cervical cancer were advancing age, STI, abortion, sitting on a hot chair or stone, vaginal bleeding and discharge, lack of personal hygiene, MSP, parity, and holding urine for long periods. Some believe that feeding the breast and working load were risk factors for cervical cancer. And also, none of the participants mentioned HPV as the cause of cervical cancer^39^. This is consistent with the findings of similar studies carried out in Nigeria^40^. Although it was proclaimed a health priority of the Ethiopian government^16^, knowledge and awareness about cervical cancer are low in Ethiopian communities. Therefore, education on women's health problems seems to be an important component of awareness of cervical cancer to make informed decisions^41^.

## Conclusions

This population-based study showed a moderate prevalence of human papillomavirus (HPV) infection among women who attended cervical cancer screening at the FGAE clinic in Addis Ababa (9.72%).These results highlight the need to develop a national cervical screening and HPV vaccination program to reduce the burden of cervical cancer in Addis Ababa. Replication of this study in rural Ethiopian populations is highly required. Therefore, we recommend that women who have started sexual intercourse earlier and who gave birth before 18 years should be given priority for cervical screening. HPV genotyping should also be studied to become aware and gain more information to avoid consequences in the future.

### Supplementary Information

Supplementary Information 1.

Supplementary Information 2.

## Data Availability

Not applicable. However, raw data have been provided in form of a Supplementary file.
