# Supplementary material for: Prevalence of human papillomavirus infection and associated factors among women attending cervical cancer screening in setting of Addis Ababa, Ethiopia
Source: Sci Rep. 2024 Feb 19;14:4053. doi: 10.1038/s41598-024-54754-x (PMC10876560; doi:10.1038/s41598-024-54754-x)
Supplement: Supplementary file 1 — Supplementary Information 1. [file 41598_2024_54754_MOESM1_ESM.pdf]

Gel Doc Result

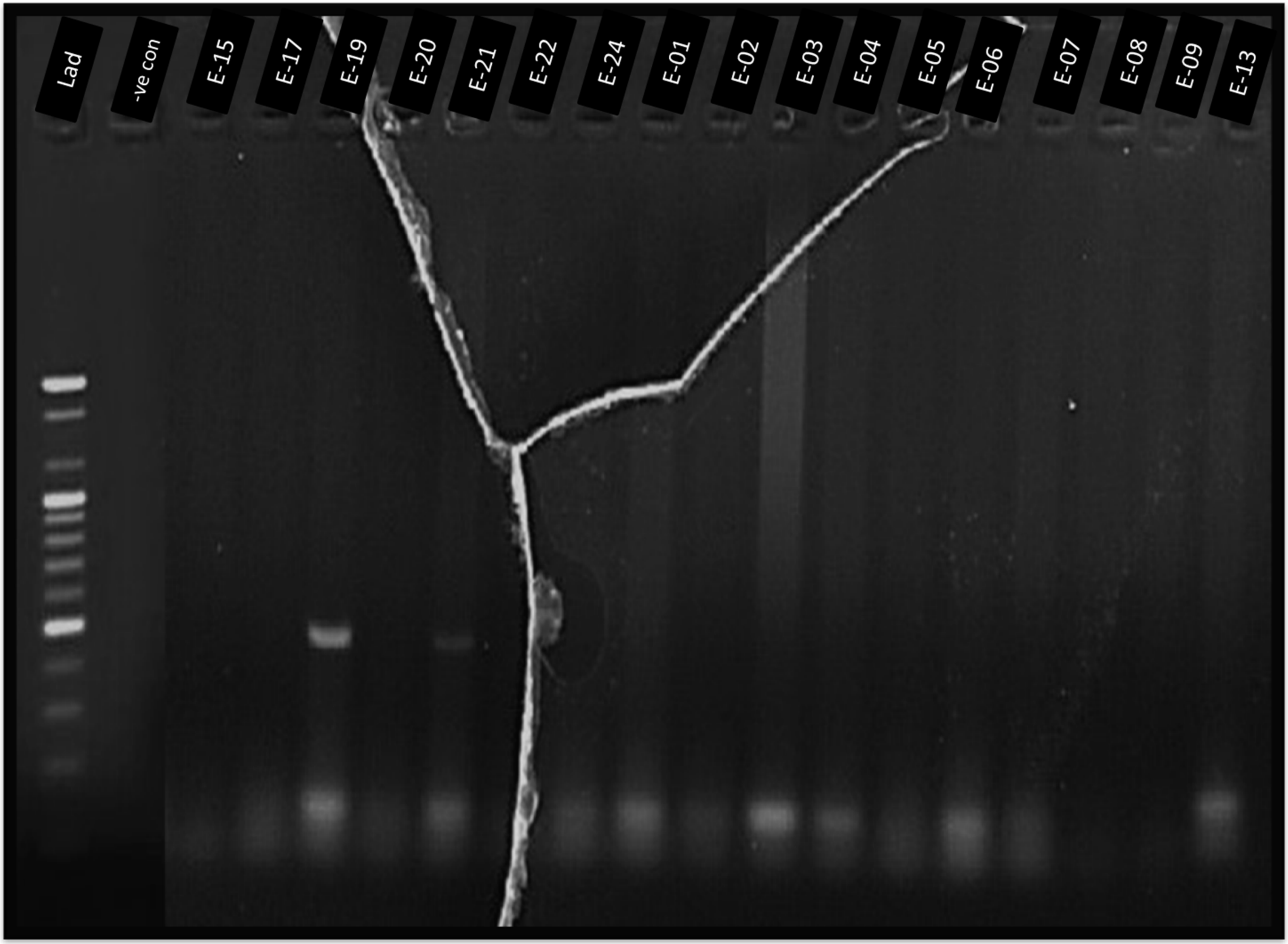
Lad
-ve con
E-15
E-17
E-19
E-20
E-21
E-22
E-24
E-01
E-02
E-03
E-04
E-05
E-06
E-07
E-08
E-09
E-13

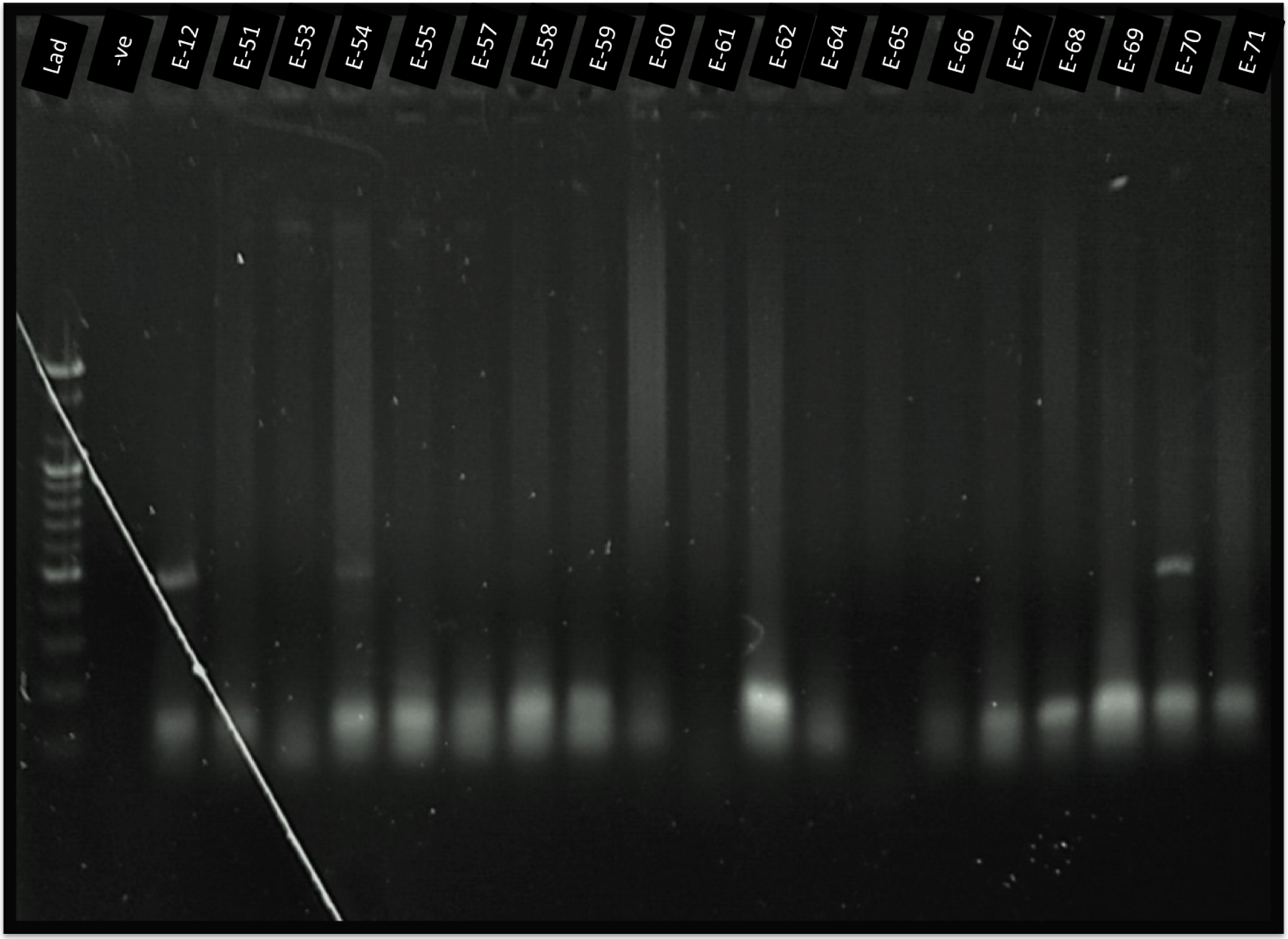
Lad
-ve
E-12
E-51
E-53
E-54
E-55
E-57
E-58
E-59
E-60
E-61
E-62
E-64
E-65
E-66
E-67
E-68
E-69
E-70
E-71

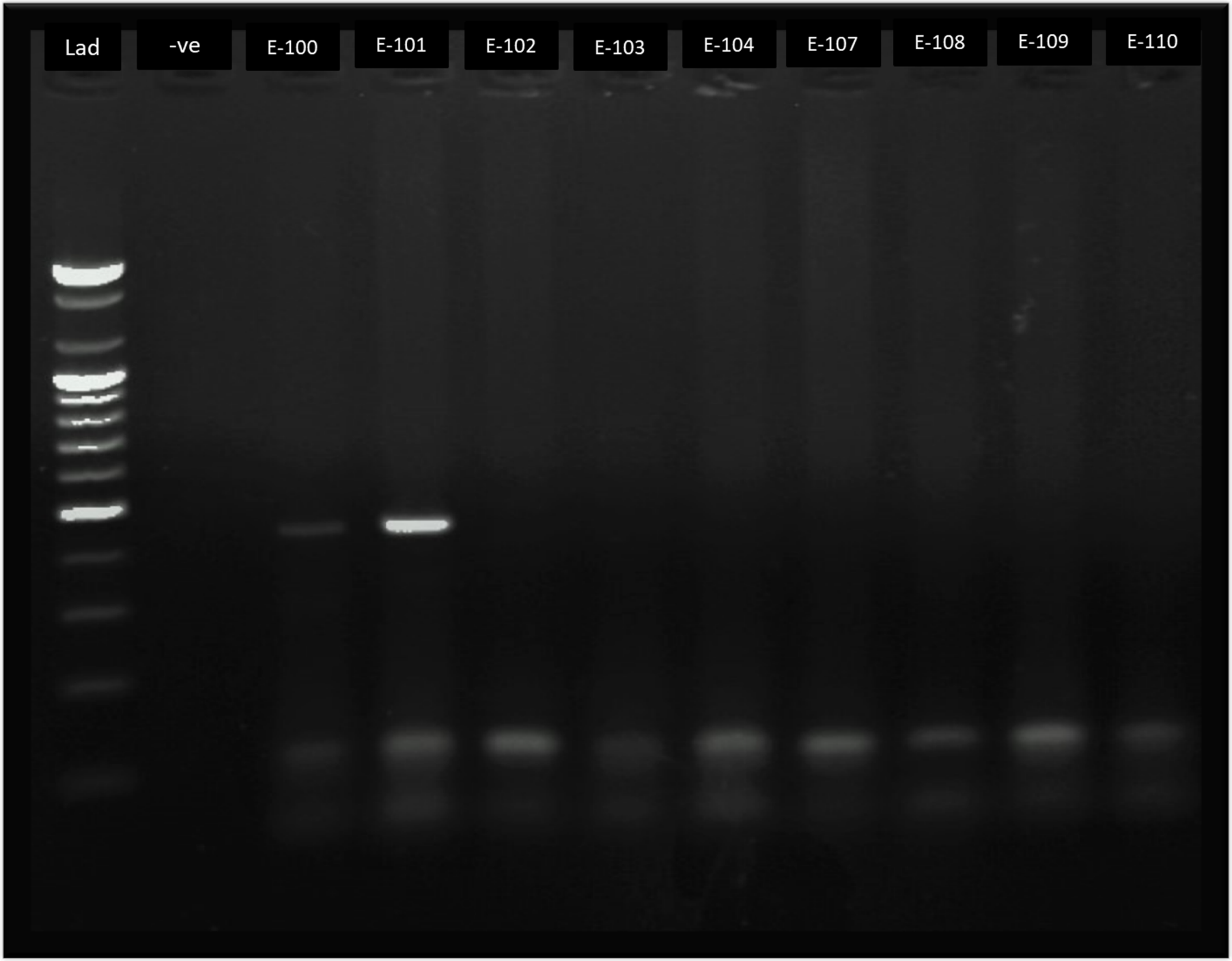
Lad
-ve
E-100
E-101
E-102
E-103
E-104
E-107
E-108
E-109
E-110

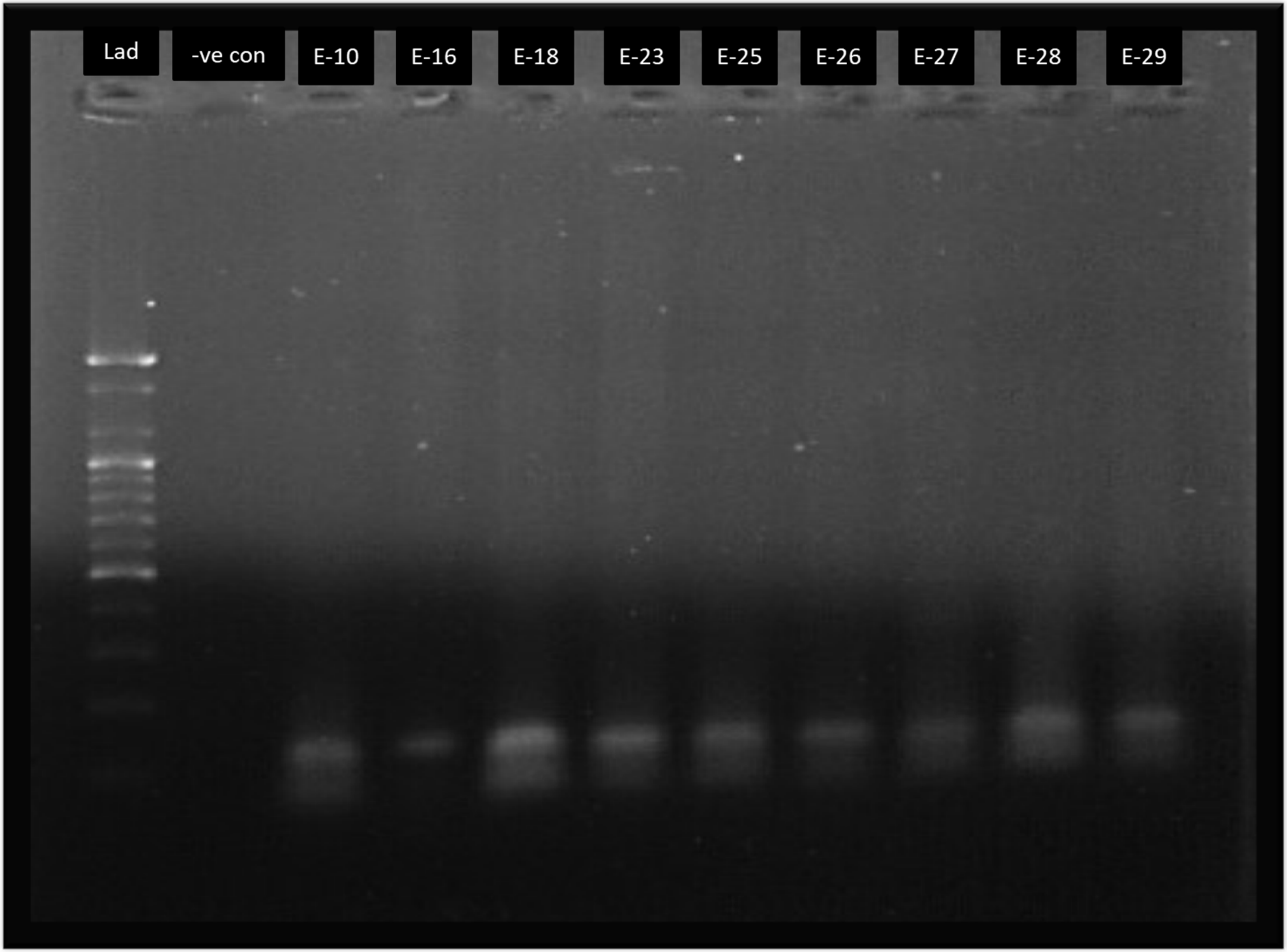
Lad
-ve con
E-10
E-16
E-18
E-23
E-25
E-26
E-27
E-28
E-29

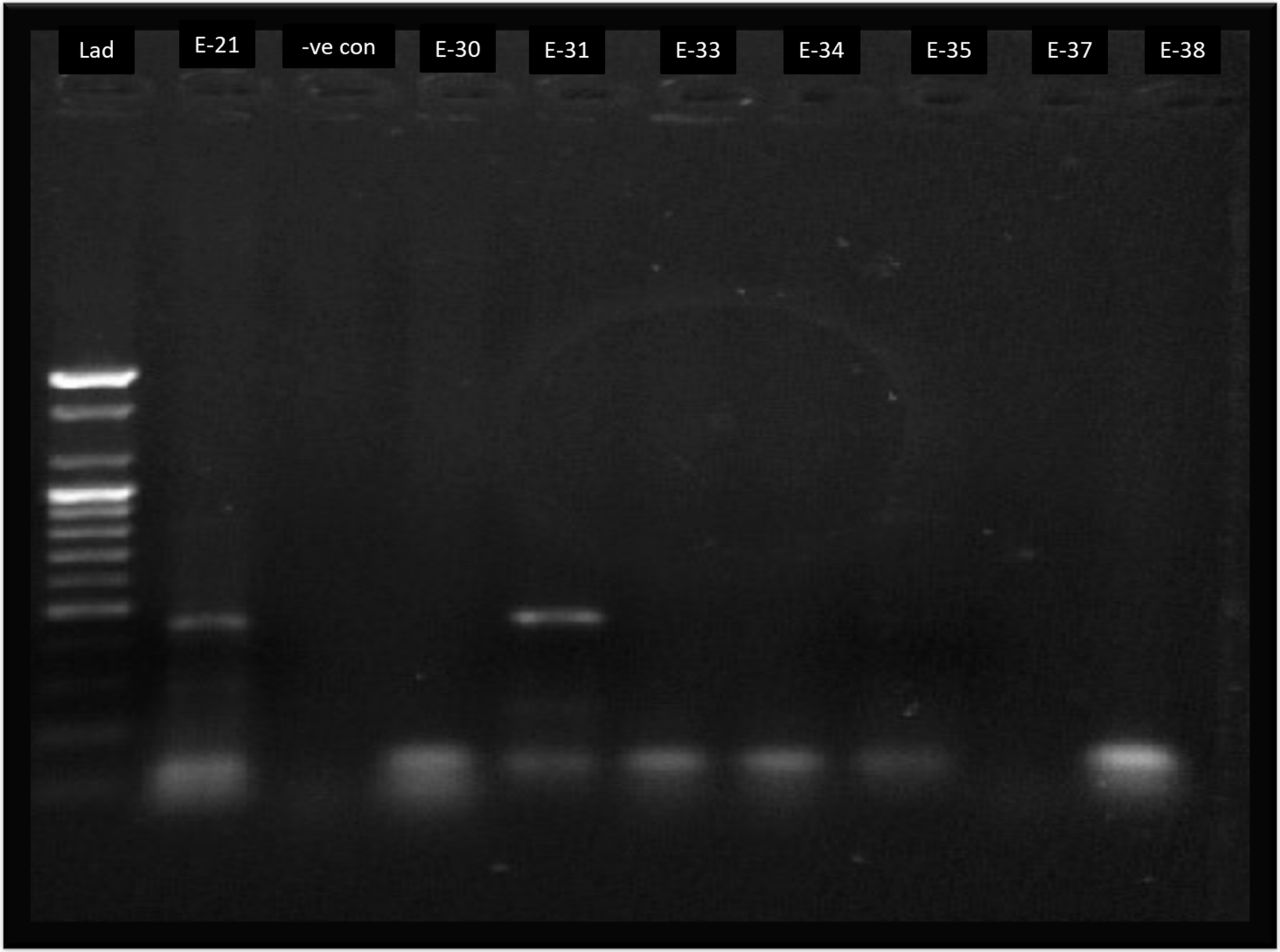
Lad
E-21
-ve con
E-30
E-31
E-33
E-34
E-35
E-37
E-38

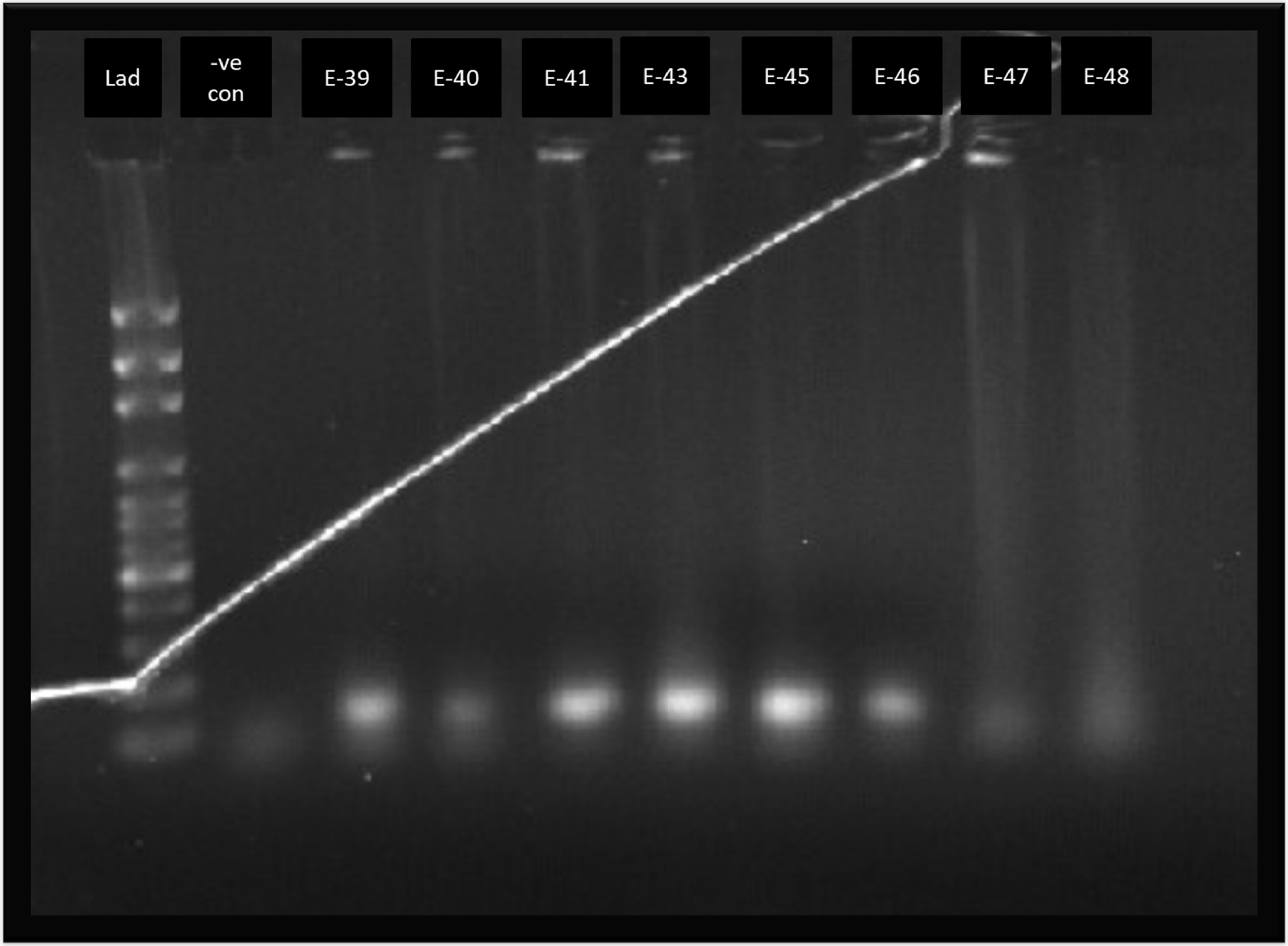
Lad
-ve con
E-39
E-40
E-41
E-43
E-45
E-46
E-47
E-48

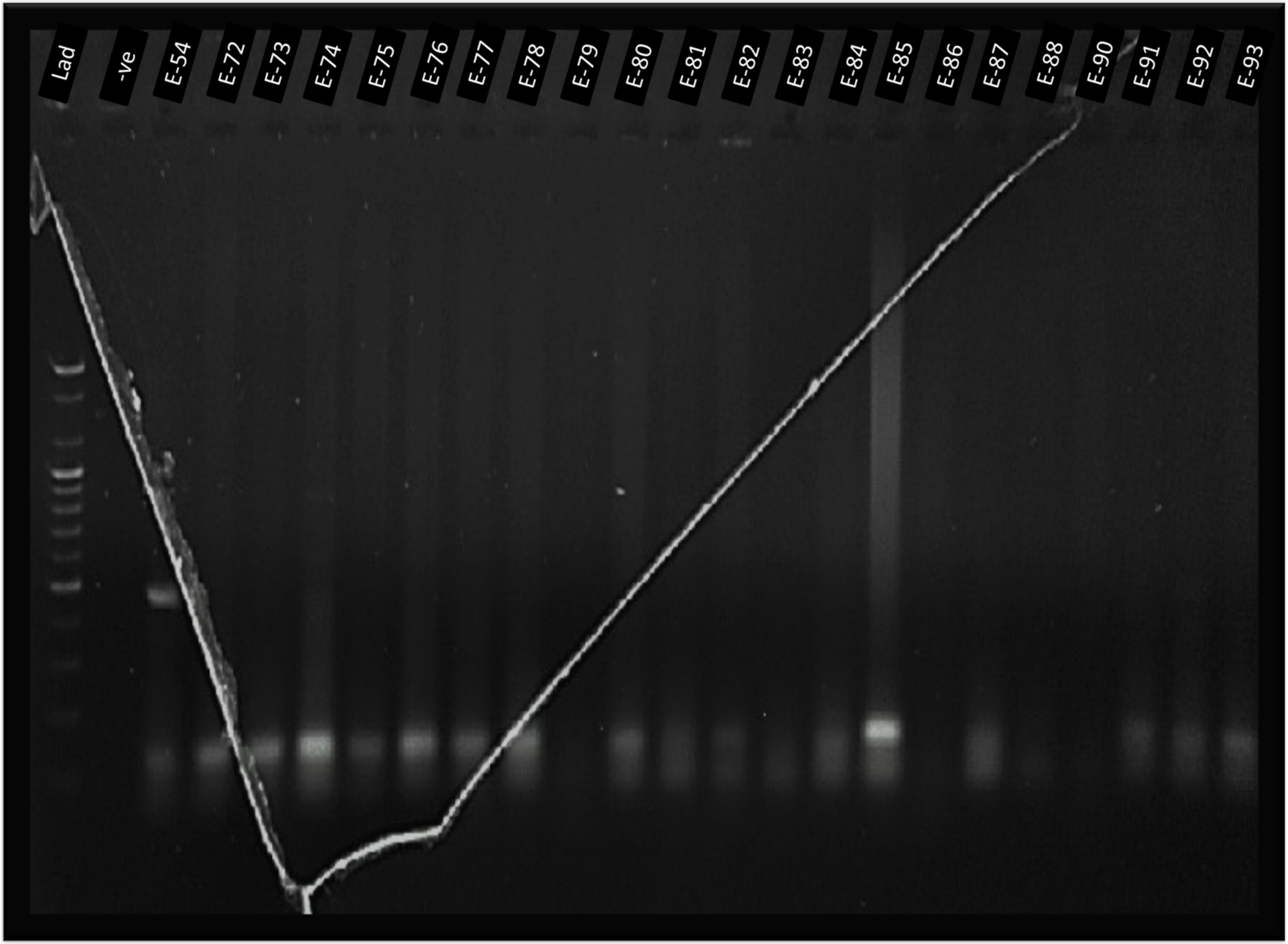
Lad
-ve
E-54
E-72
E-73
E-74
E-75
E-76
E-77
E-78
E-79
E-80
E-81
E-82
E-83
E-84
E-85
E-86
E-87
E-88
E-90
E-91
E-92
E-93

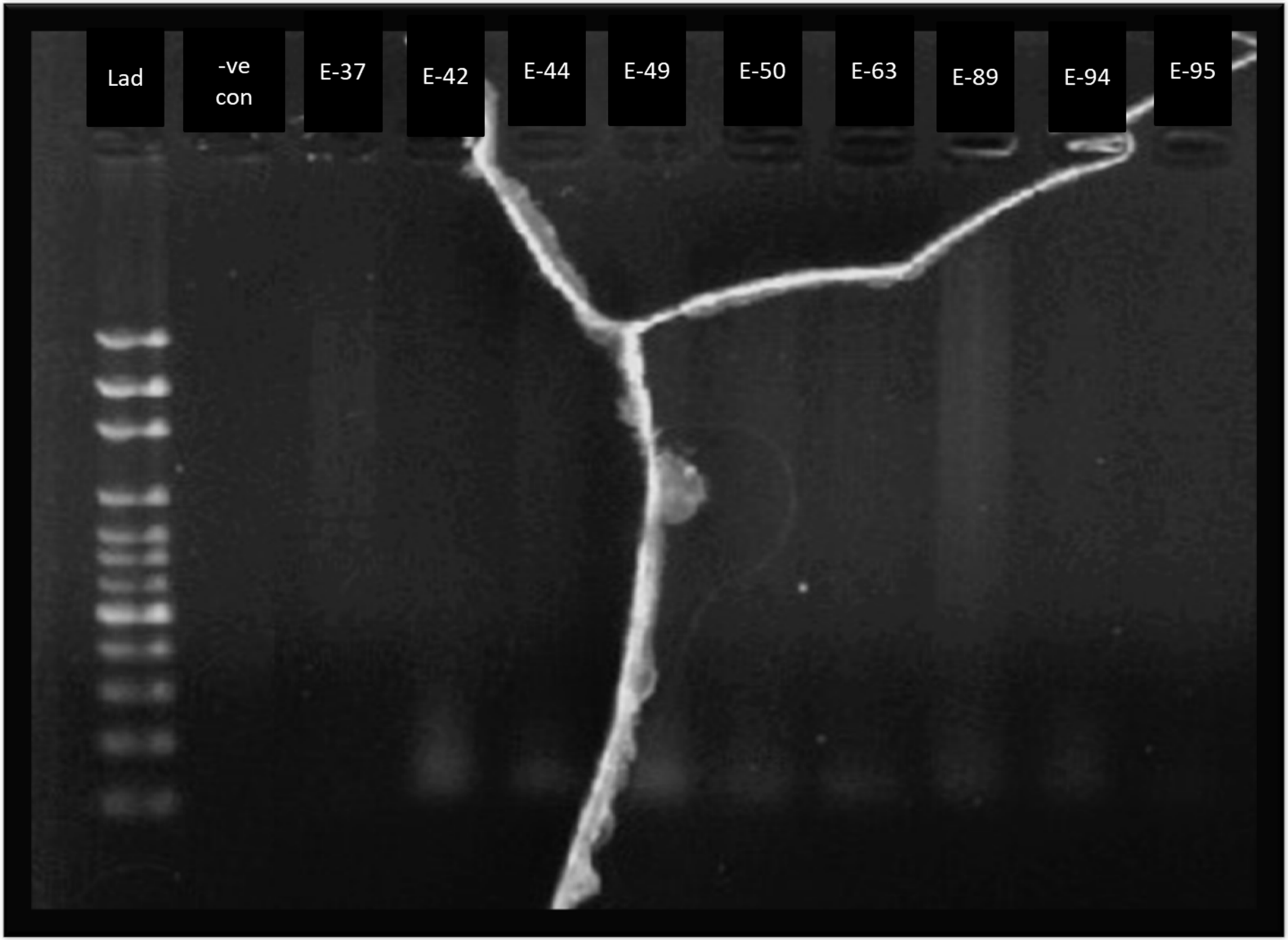
Lad
-ve con
E-37
E-42
E-44
E-49
E-50
E-63
E-89
E-94
E-95

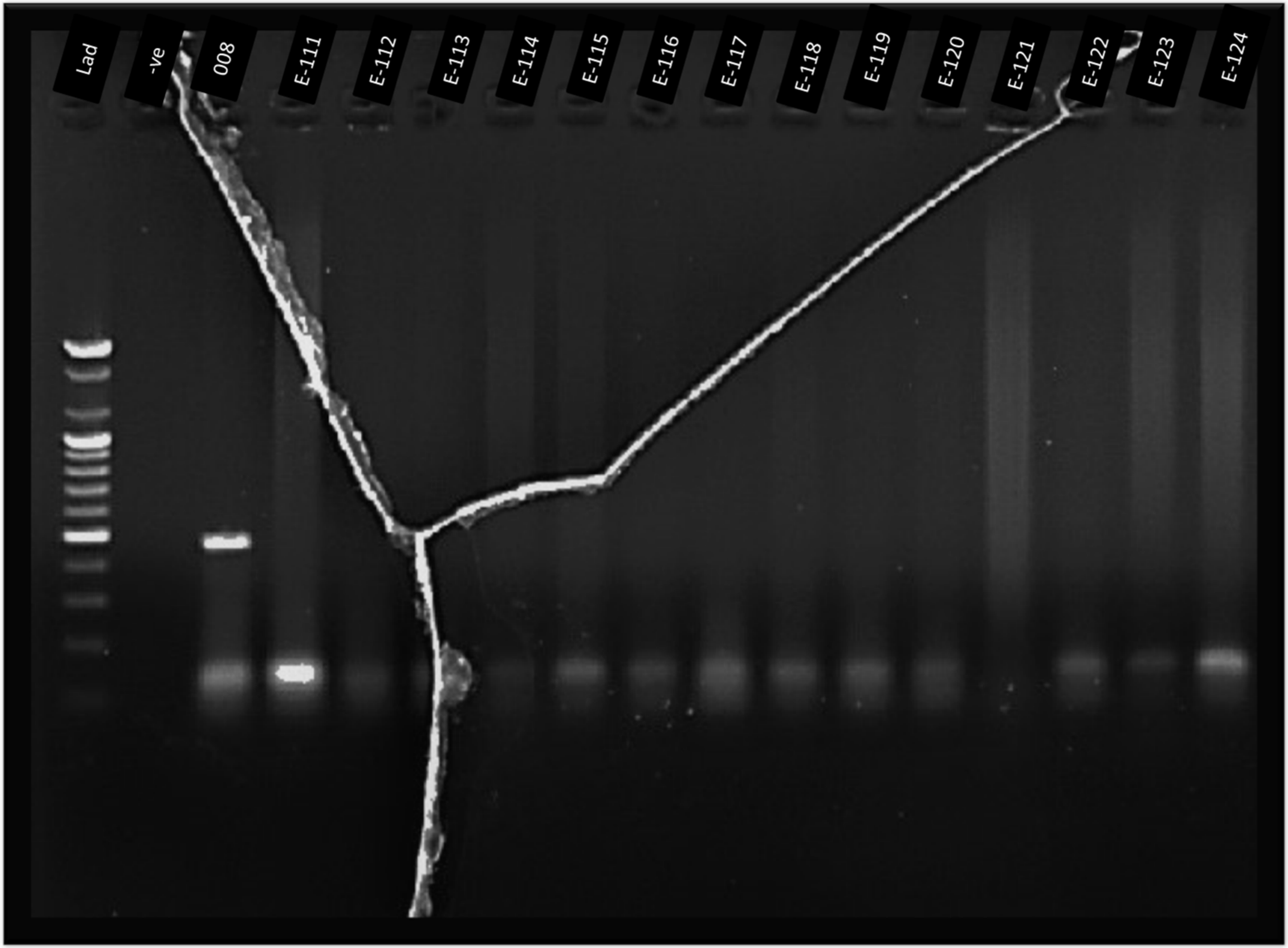
Lad
-ve
008
E-111
E-112
E-113
E-114
E-115
E-116
E-117
E-118
E-119
E-120
E-121
E-122
E-123
E-124

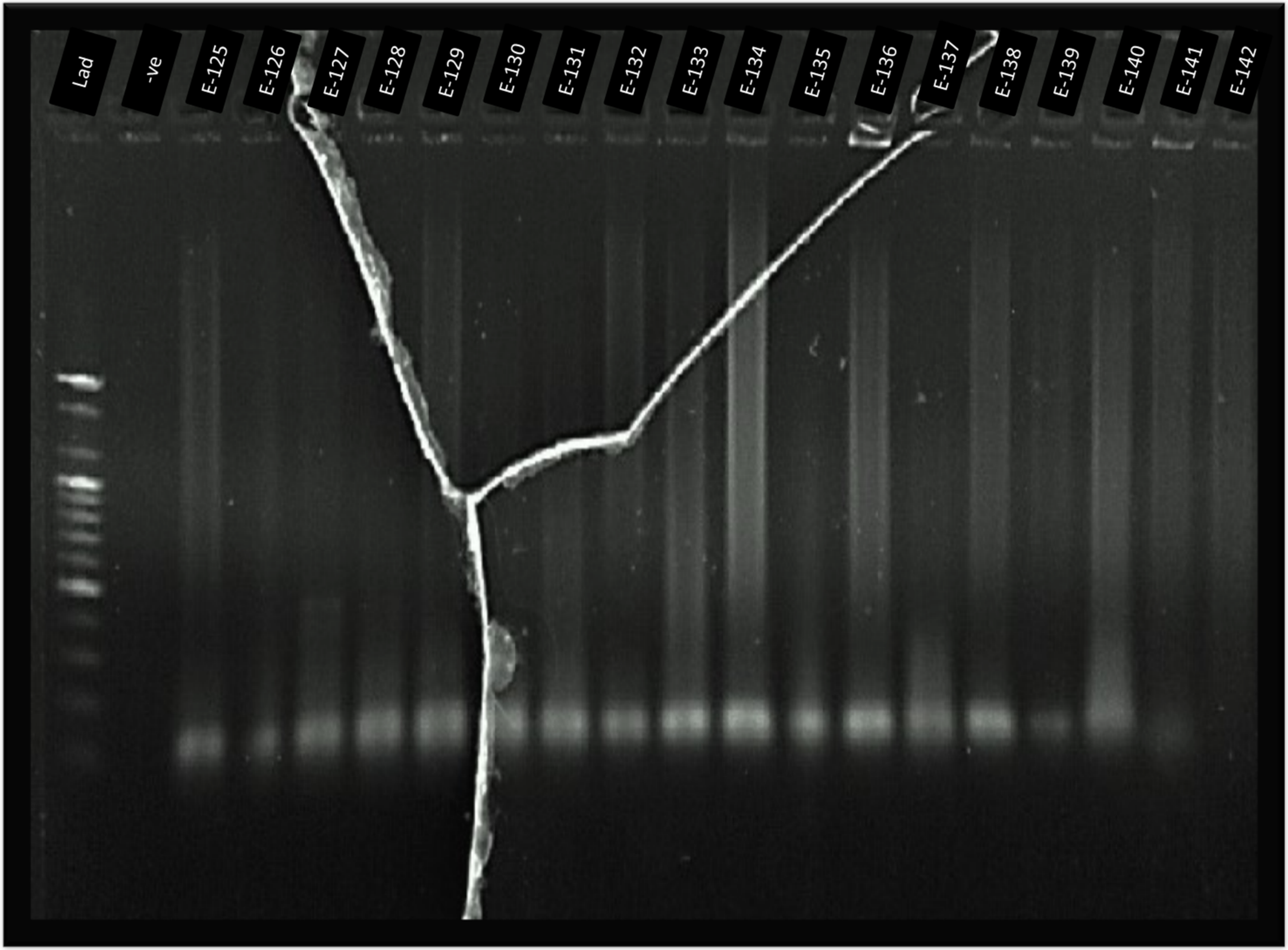
Lad
-ve
E-125
E-126
E-127
E-128
E-129
E-130
E-131
E-132
E-133
E-134
E-135
E-136
E-137
E-138
E-139
E-140
E-141
E-142

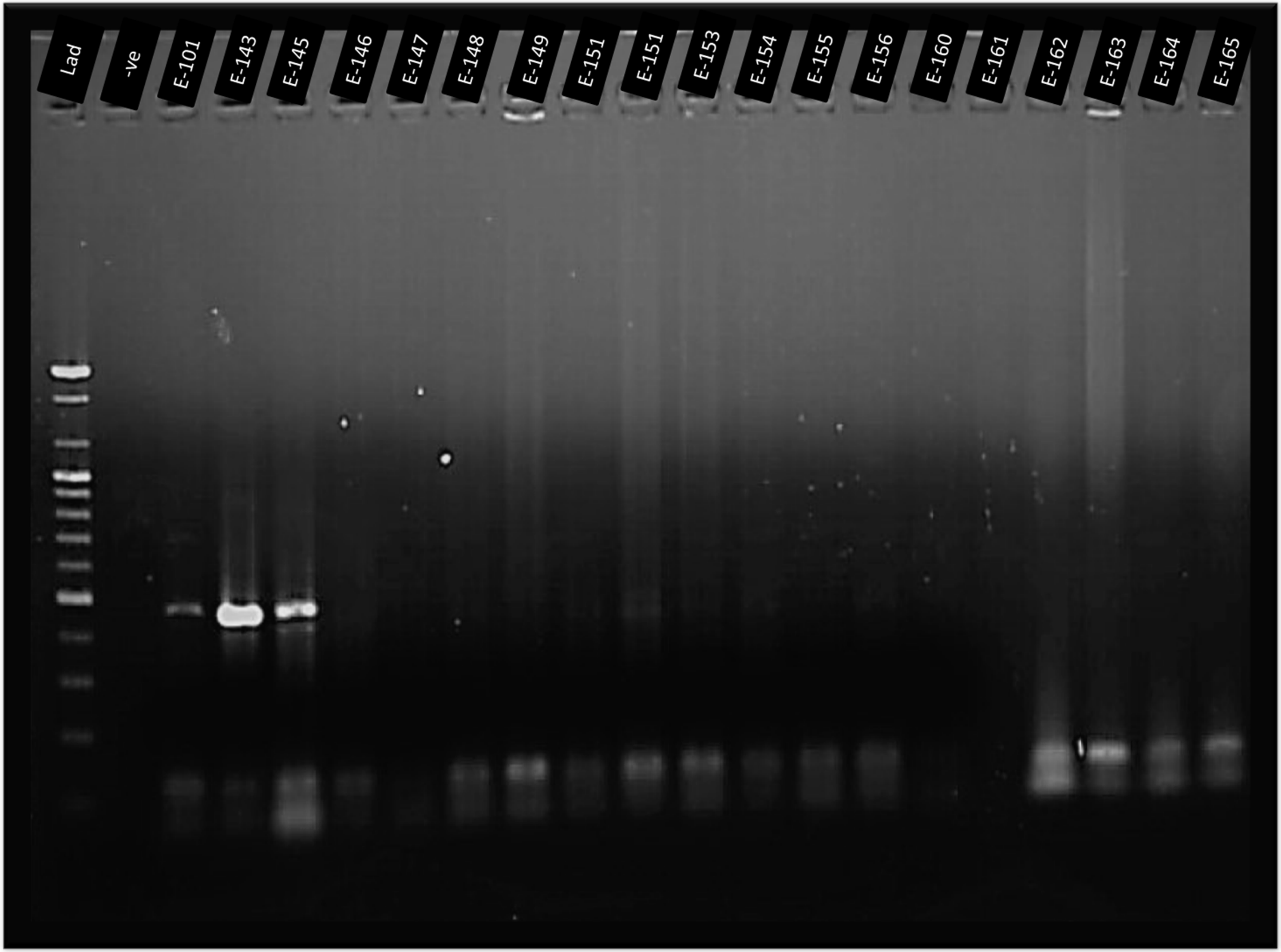
Lad
-ve
E-101
E-143
E-145
E-146
E-147
E-148
E-149
E-151
E-151
E-153
E-154
E-155
E-156
E-160
E-161
E-162
E-163
E-164
E-165

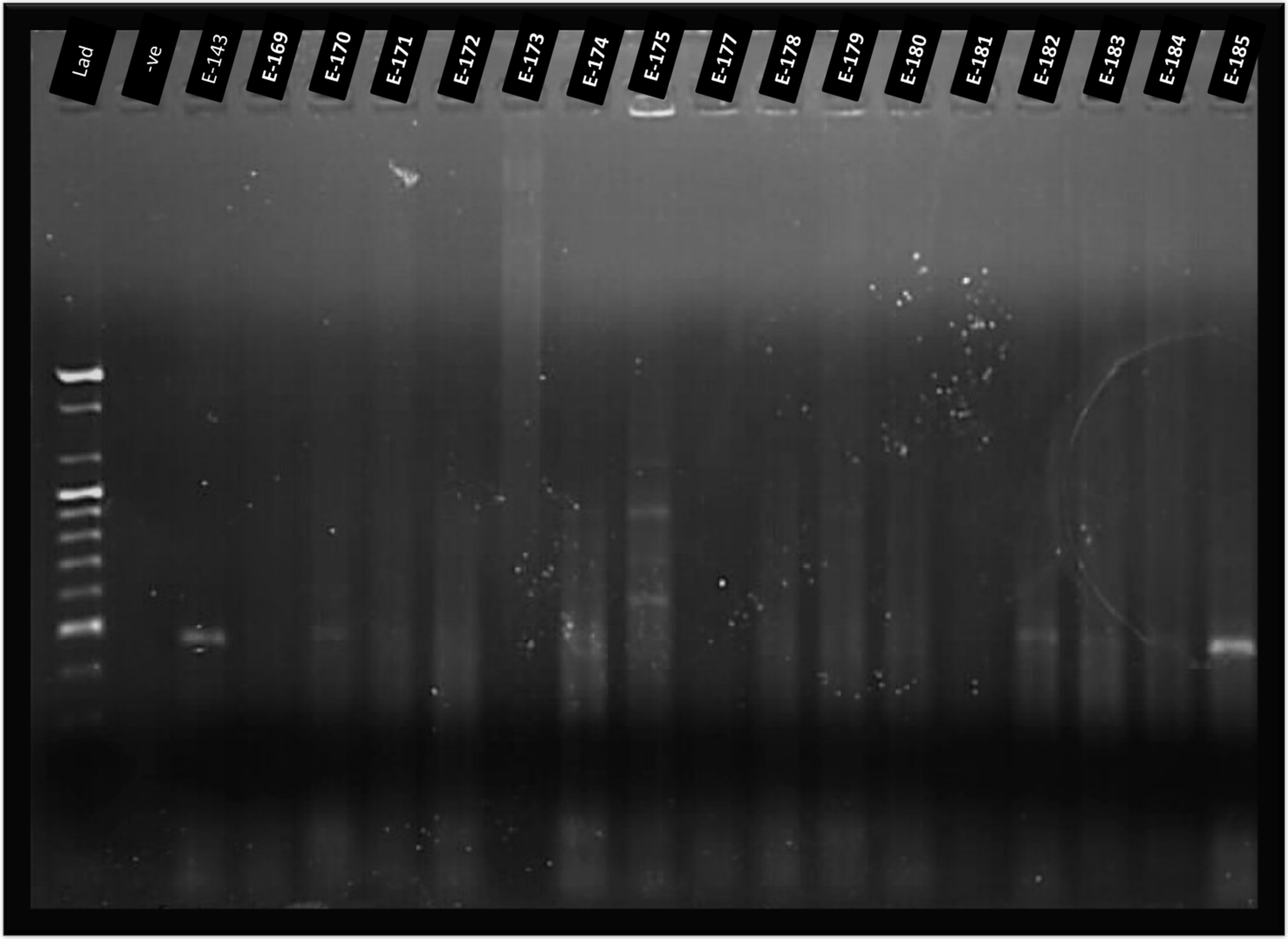
Lad
-ve
E-143
E-169
E-170
E-171
E-172
E-173
E-174
E-175
E-177
E-178
E-179
E-180
E-181
E-182
E-183
E-184
E-185

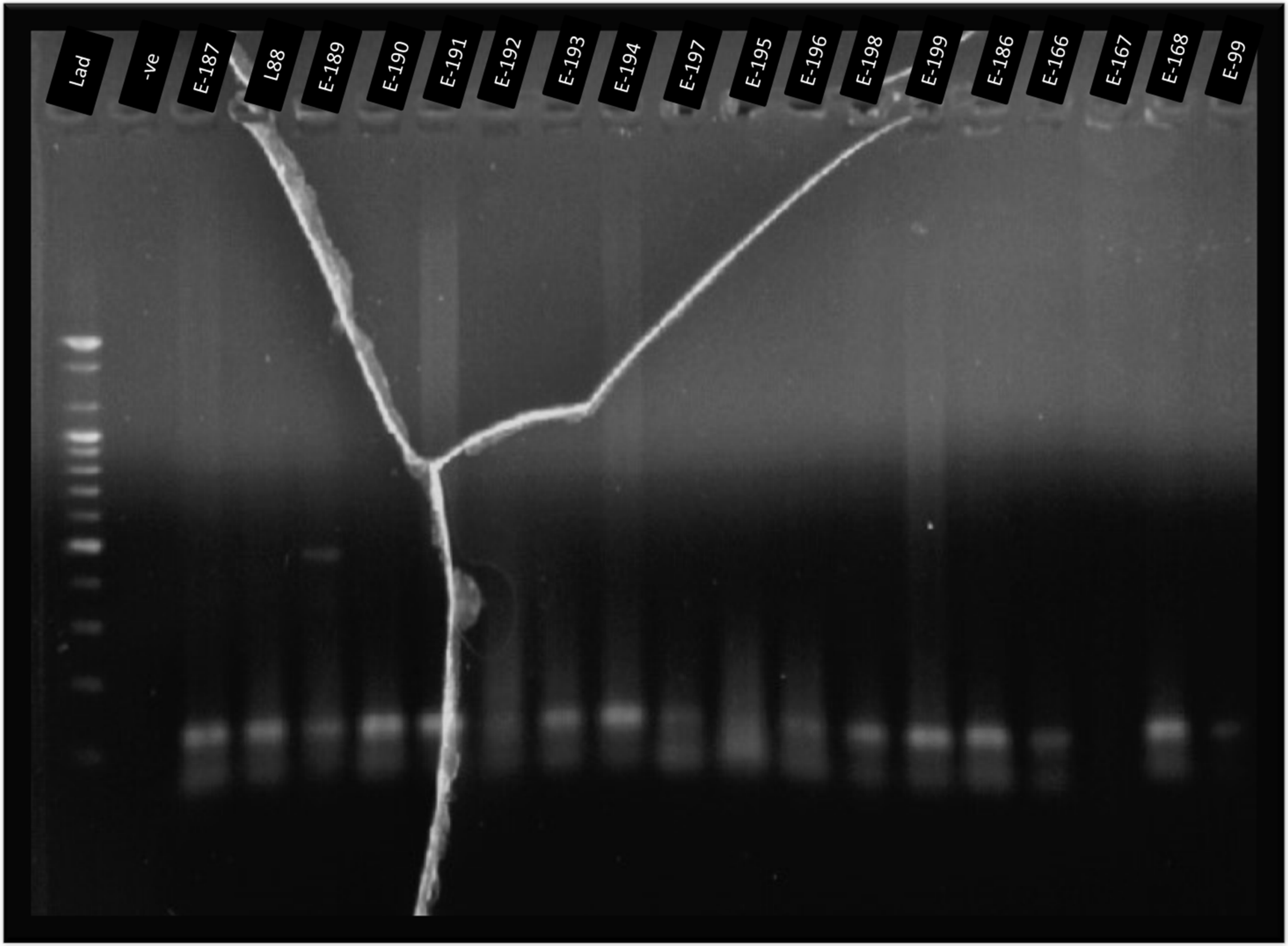
Lad
-ve
E-187
L88
E-189
E-190
E-191
E-192
E-193
E-194
E-197
E-195
E-196
E-198
E-199
E-186
E-166
E-167
E-168
E-99

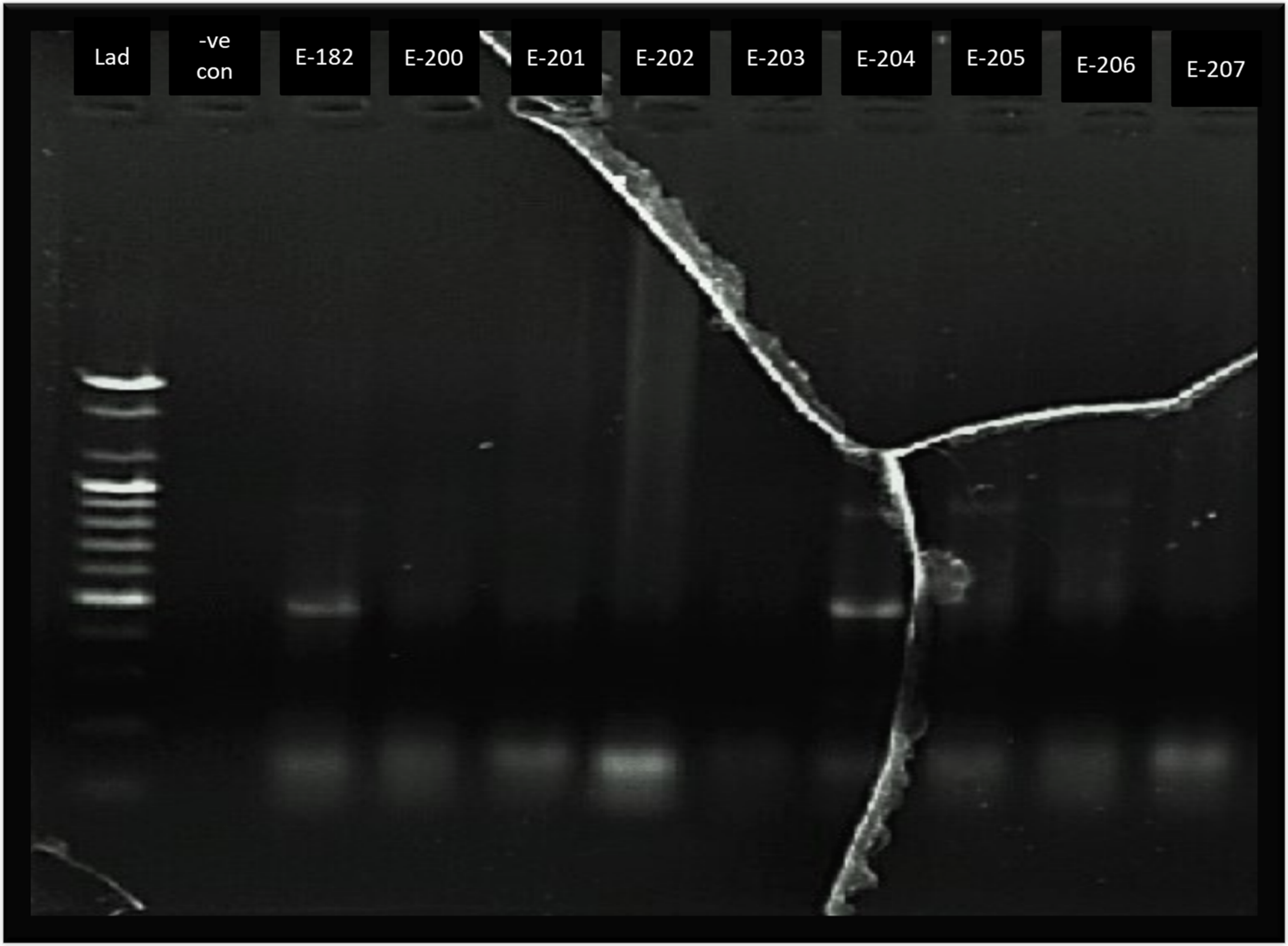
Lad
-ve con
E-182
E-200
E-201
E-202
E-203
E-204
E-205
E-206
E-207

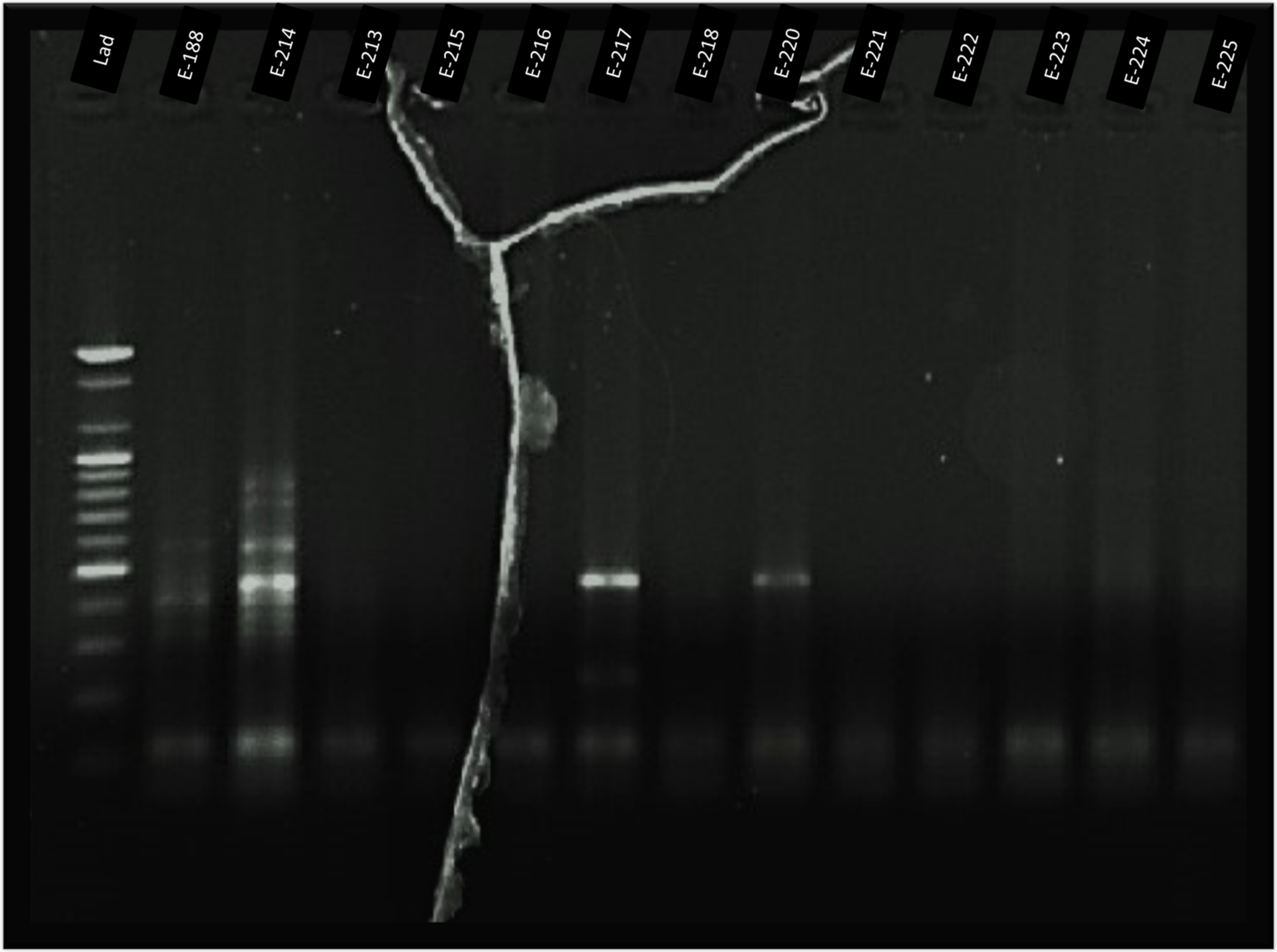
Lad
E-188
E-214
E-213
E-215
E-216
E-217
E-218
E-220
E-221
E-222
E-223
E-224
E-225

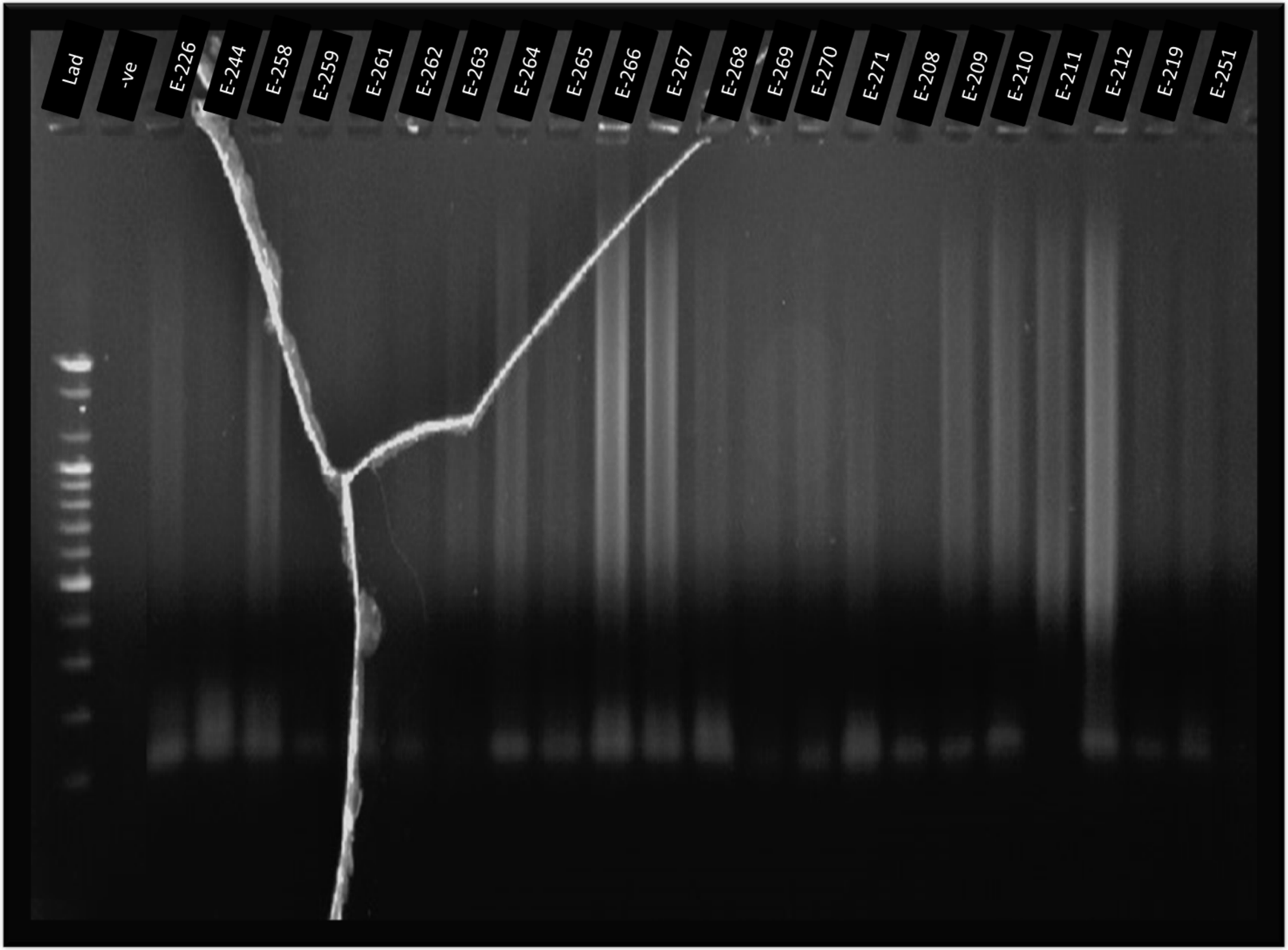
Lad
-ve
E-226
E-244
E-258
E-259
E-261
E-262
E-263
E-264
E-265
E-266
E-267
E-268
E-269
E-270
E-271
E-208
E-209
E-210
E-211
E-212
E-219
E-251

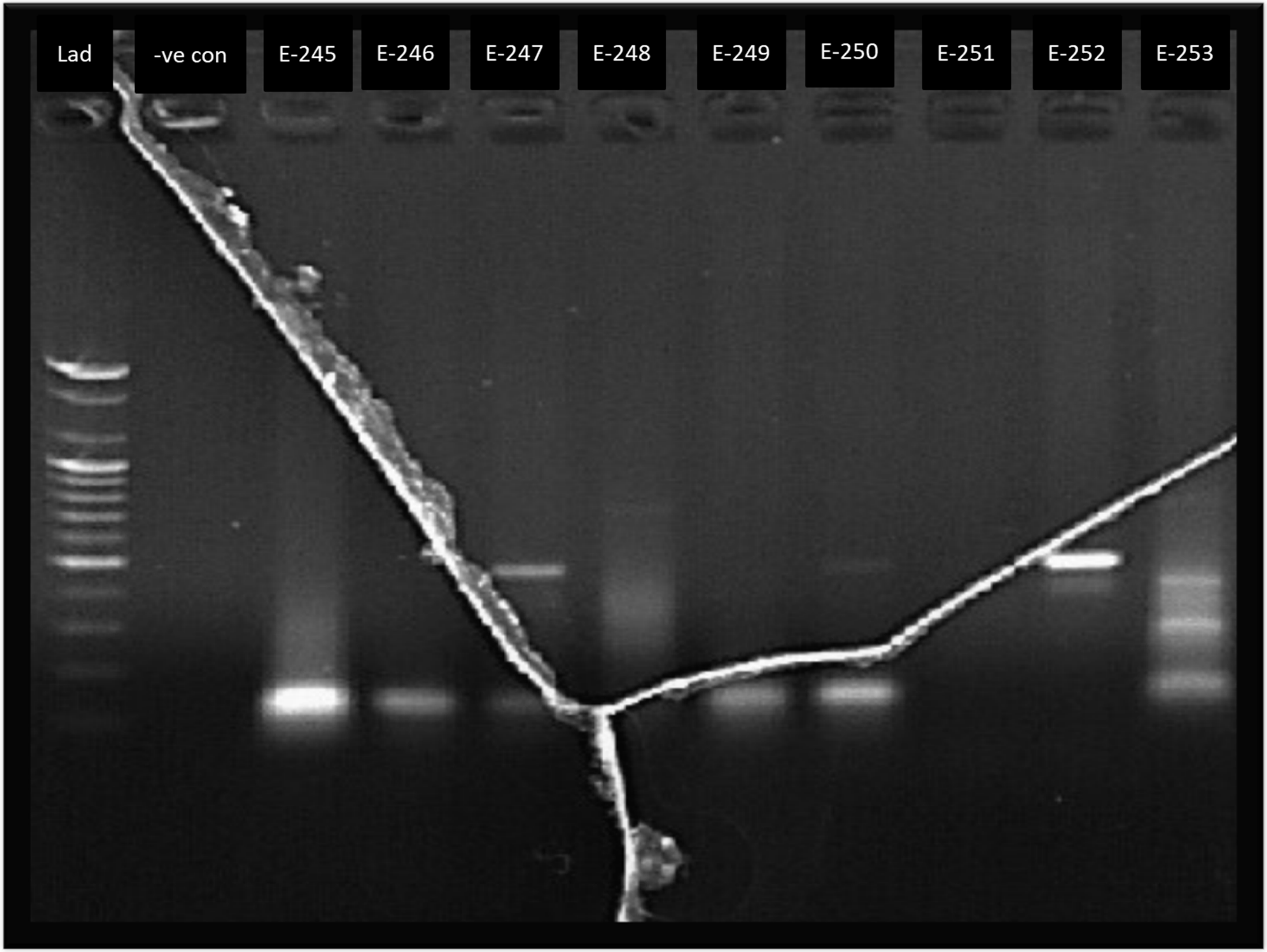
Lad
-ve con
E-245
E-246
E-247
E-248
E-249
E-250
E-251
E-252
E-253

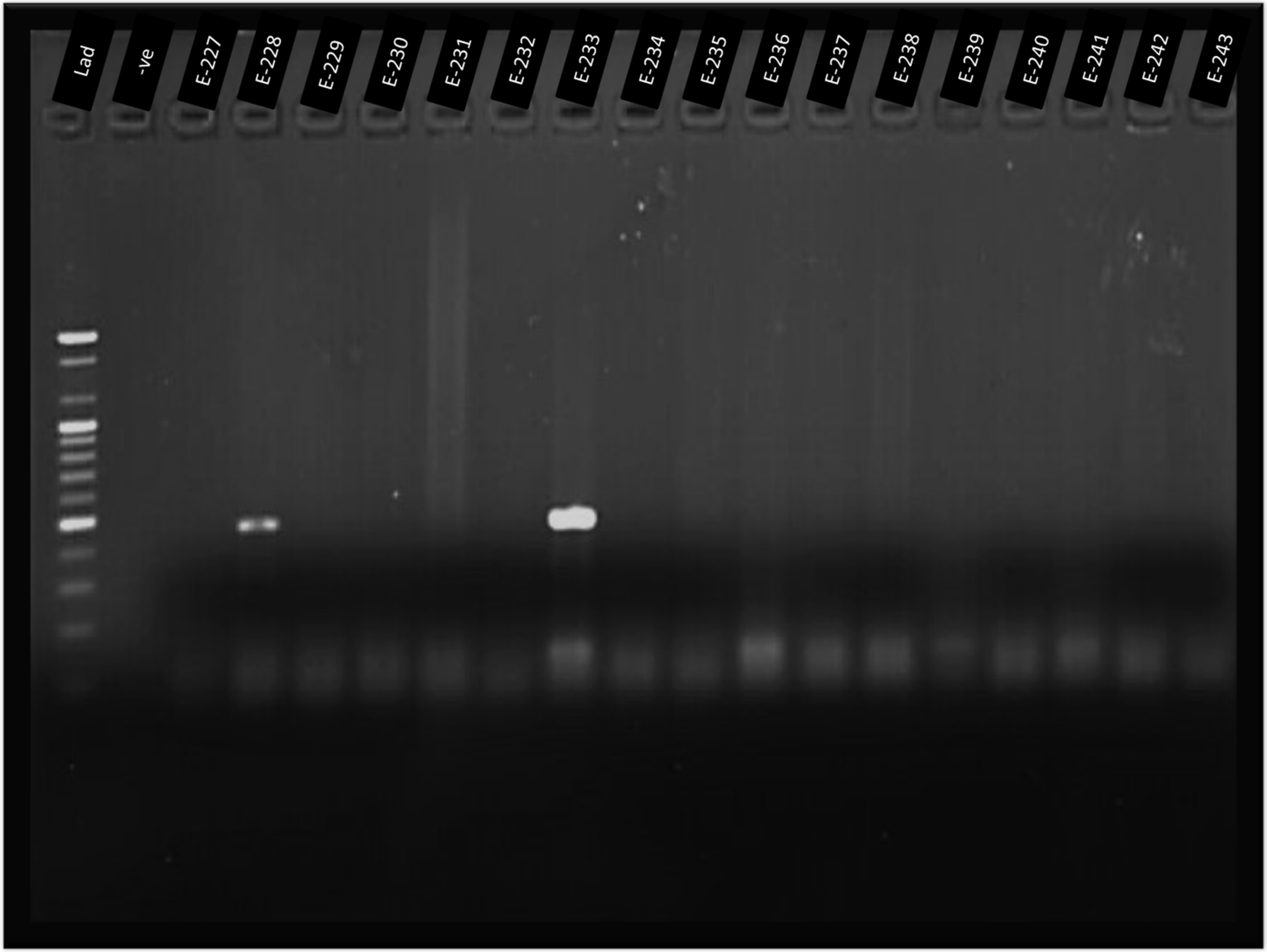
Lad
-ve
E-227
E-228
E-229
E-230
E-231
E-232
E-233
E-234
E-235
E-236
E-237
E-238
E-239
E-240
E-241
E-242
E-243
