# Supplementary material for: Prevalence of human papillomavirus infection and associated factors among women attending cervical cancer screening in setting of Addis Ababa, Ethiopia
Source: Sci Rep. 2024 Feb 19;14:4053. doi: 10.1038/s41598-024-54754-x (PMC10876560; doi:10.1038/s41598-024-54754-x)
Supplement: Supplementary file 2 — Supplementary Information 2. [file 41598_2024_54754_MOESM2_ESM.pdf]

Supplementary File 2

Questionnaire (English version)

DILLA UNIVERSITY

SCHOOL OF GRADUATE STUDIES

COLLEGE OF NATURAL AND COMPUTATIONAL SCIENCES

DEPARTMENT OF BIOLOGY

Questionnaire

Introduction: Dear respondents, this questionnaire is prepared as an instrument to conduct an academic research on the topic entitled ‗Human papillomavirus and *Chlamydial trachomatis* prevalence among women attending cervical cancer screening at Family Guidance Association of Ethiopia, Addis Ababa clinic. The information you will provide is very useful for successful accomplishment of this study. It is your full right to participate, withdraw or refuse in the participation of this study. All of your answers will be kept confidential. I will assure you that there will be no problem regarding with the participation of this study. Therefore you are kindly requested to provide consent of participation.

Would you be willing to answer the questions?

Yes ☐ 2. No ☐

Thank you for giving your time and agreeing. If there are any questions that you want to ask, please do not hesitate to ask at any time.

No	Questioner	Answer	
**I-**	**Socio-demographics data**		
1	How old are you?	16 – 25	
		26 – 35	

		Above 36	
2	What is your marital status?	Married	
		Single	
		Divorced	
		Widowed	
		Other፡	
3.	What is the highest standard of education attained?	None	
		Read and write	
		Primary (1-8)	
		Grade 9-12	
		Certificate	
		Diploma	
		1st Degree and above	
4	How can you describe your employment status at present?	Employed full-time	
		Employed part-time	
		Unemployed	
		Student	
		Other፡	
4A	How much do you earn per month?	500 and less Birr	
		501-1500 Birr	
		1501-3000 Birr	
		More than 3000 Birr	

**II- Behavioral characteristics data**			
5	Have you ever used alcohol?	Yes	
		No	
5A	How often did you have at least one drink of any alcoholic beverage?	Per days	
		Per weak	
		Per month	
		During festival	
6	Have you ever used any form of tobacco (cigarettes, pipes, cigars, chew, snuff)?	Yes	
		No	
6A	If answer to Q. 6 is ―yes‖ how many years have you smoked tobacco?	3- 6 Month	
		1-3 year	
		4-5 Year	
		More than 5 year	
6B	Do you smoke tobacco now?	Yes	
		No	
**III- Reproductive characteristics data**			
7	Have you ever had a sexual intercourse?	Yes	
		No	
7A	How old were you when you first had sex?	12-17	
		18-23	
		Above 24	
7B	In your life, what is the number of men	1	

	with whom you have had sex?	2 – 4	
		More than 4	
8	Are you on any method of contraception?	Yes	
		No	
8A	If answer to Q. 8 is –yes‖ specify method.	Pills	
		Injectable Depo	
		Implant	
		Condom	
		IUD (loop)	
		Levonelle/ emergency contraceptive	
		Other:	
9	Have you ever been pregnant?	Yes	
		No	
9A	If answer to Q.9 –yes‖. How old were you when you first get pregnant?	12-17	
		18-23	
		Above 24	
10	Did you have any abortion case?	Yes	
		No	
10A	If answer to Q. 10 is –yes‖ At what trimester?	1-3 first trimester	
		4-6 Second trimester	
		7-9 Third trimester	

11	Have you ever screened for cervical cancer?	Yes	
		No	
12	Why did you want to be screened?	My doctor ordered the screening	
		I suspect something is wrong with my cervix or womb	
		I observed some signs and symptoms	
		I just wanted to know my status	
		Other reason :	
13	What do you think are risk factor for cervical cancer?		

Table: HPV prevalence among socio-demographics variables

**Characteristics**		**HPV**		**p- value**
		**Negative**	**Positive**	
**AGE**	16 – 25	2	0	0.8875
	26 – 35	30	3	
	36 and Above	191	21	
**Marital status**	Divorced	34	4	0.7825
	Married	141	14	
	Single	17	3	
	Widowed	31	3	

**Educational status**	1st Degree and above		26	1	0.9374
	Diploma		28	2	
	Certificate		11	1	
	Grade 9-12		65	8	
	Primary (1-8)		54	7	
	Read and write		9	1	
	Illiterate		30	4	
**Employment status**	Employed full-time		76	6	0.5456
	Employed part-time		25	3	
	Student		4	1	
	Unemployed		118	4	
	Earn per month	More than 3000 Birr	145	14	0.873
		1501-3000 Birr	36	5	
		501-1500 Birr	31	4	
		500 and less Birr	11	1	

DNA extraction protocol used in the study

DNA isolation protocol from cotton swab

1. Immerse the dried cotton swab in 1.2 mL lysis solution (Lysis solution = 10 mM trisHCL, 5 mM ethelyne diamine tetra acetate (EDTA) and 5% sodium dodisyl sulphate at pH 8.0 containing 12 µL of 20mg/mL proteinase K
2. Incubate at 55oC for 6 hours, (and keep it at room temperature overnight if necessary)
3. Remove the cotton swab
4. Add 650 µL cold solution of 8 M ammonium acetate with 1 mM EDTA and mix by gently vortexing
5. Chill on ice for about 10 minutes
6. Centrifuge at 6000 rpm for 20 minutes
7. Transfer 750 µL of the supernatant in to two clean 1.5 mL microtubes containing 750 µL cold isopropanol with wide bore pipette tips and mix by inverting gently several times (~20 times)
8. Centrifuge at 13000rpm for 10minutes and pour off the supernatant and invert each tube and leave to drain on clean absorbent paper
9. Add 1mL 90% ethanol and mix by inverting gently several times
10. Centrifuge at 13000 rpm for 10 minutes and pour off the ethanol and invert each tube and leave to drain on clean absorbent paper
11. Re-suspend the pellet in 100 µL 1XTE buffer
